# Reactivity and Stability of Reduced Ir-Weight TiO_2_-Supported Oxygen Evolution Catalysts for Proton Exchange Membrane (PEM) Water Electrolyzer Anodes

**DOI:** 10.1021/jacs.4c07002

**Published:** 2024-11-11

**Authors:** Hoang Phi Tran, Hong Nhan Nong, Matej Zlatar, Aram Yoon, Uta Hejral, Martina Rüscher, Janis Timoshenko, Sören Selve, Dirk Berger, Matthias Kroschel, Malte Klingenhof, Benjamin Paul, Sebastian Möhle, Kerolus Nasser Nagi Nasralla, Daniel Escalera-López, Arno Bergmann, Serhiy Cherevko, Beatriz Roldan Cuenya, Peter Strasser

**Affiliations:** † Department of Chemistry, Chemical Engineering Division, The Electrochemical Energy, Catalysis and Materials Science Laboratory, Technische Universität Berlin, Straße des 17. Juni 124, 10623 Berlin, Germany; ‡ Department of Chemical Engineering, Faculty of Physics and Chemical Engineering, Le Quy Don Technical University, 236 Hoang Quoc Viet, Bac Tu Liem District, Hanoi 100000, Vietnam; § Forschungszentrum Jülich GmbH, Helmholtz-Institute Erlangen-Nürnberg for Renewable Energy (IET-2), Cauerstraße 1, 91058 Erlangen, Germany; ∥ Department of Chemical and Biological Engineering, Friedrich-Alexander-Universität Erlangen-Nürnberg, Egerlandstr. 3, 91058 Erlangen, Germany; ⊥ Department of Interface Science, Fritz-Haber-Institute of the Max-Planck-Society, Faradayweg 4-6, 14195 Berlin, Germany; # Center for Electron Microscopy (ZELMI), Technische Universität Berlin, D-10623 Berlin, Germany

## Abstract

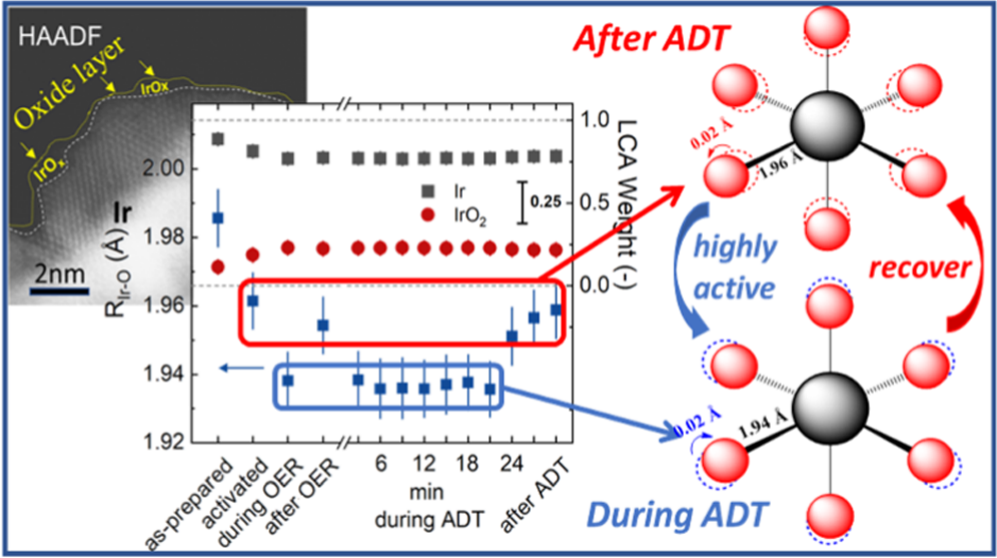

Reducing the iridium demand in Proton Exchange Membrane Water Electrolyzers (PEM WE) is a critical priority for the green hydrogen industry. This study reports the discovery of a TiO_2_-supported Ir@IrO(OH)_*x*_ core–shell nanoparticle catalyst with reduced Ir content, which exhibits superior catalytic performance for the electrochemical oxygen evolution reaction (OER) compared to a commercial reference. The TiO_2_-supported Ir@IrO(OH)_*x*_ core–shell nanoparticle configuration significantly enhances the OER Ir mass activity from 8 to approximately 150 A g_Ir_^–1^ at 1.53 V_RHE_ while reducing the iridium packing density from 1.6 to below 0.77 g_Ir_ cm^–3^. These advancements allow for viable anode layer thicknesses with lower Ir loading, reducing iridium utilization at 70% LHV from 0.42 to 0.075 g_Ir_ kW^–1^ compared to commercial IrO_2_/TiO_2_. The identification of the Ir@IrO(OH)_*x*_/TiO_2_ OER catalyst resulted from extensive HAADF-EDX microscopic analysis, operando XAS, and online ICP-MS analysis of 30–80 wt % Ir/TiO_2_ materials. These analyses established correlations among Ir weight loading, electrode electrical conductivity, electrochemical stability, and Ir mass-based OER activity. The activated Ir@IrO(OH)_*x*_/TiO_2_ catalyst–support system demonstrated an exceptionally stable morphology of supported core–shell particles, suggesting strong catalyst–support interactions (CSIs) between nanoparticles and crystalline oxide facets. Operando XAS analysis revealed the reversible evolution of significantly contracted Ir–O bond motifs with enhanced covalent character, conducive to the formation of catalytically active electrophilic O^I–^ ligand species. These findings indicate that atomic Ir surface dissolution generates Ir lattice vacancies, facilitating the emergence of electrophilic O^I–^ species under OER conditions, while CSIs promote the reversible contraction of Ir–O distances, reforming electrophilic O^I–^ and enhancing both catalytic activity and stability.

## 1 Introduction

**Figure 1:**
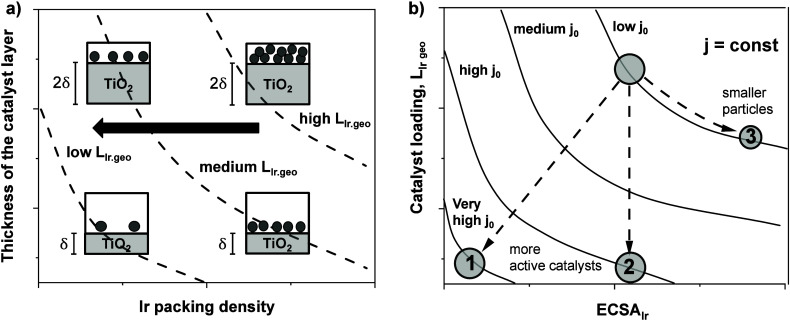
Illustration of design constrains and challenges for low Ir-loaded PEM water electrolyzer anodes. A lower Ir demand of PEM electrolyzer anodes requires lower geometric Ir loadings, L_Ir,geo_. (a) Relation between anode layer thickness and volumetric Ir packing density with dashed iso-L_Ir,geo_ curves. Arrow indicates the desired reduction in L_Ir,geo_ at constant layer thickness. (b) Qualitative relation between geometric Ir loading, L_Ir,geo_, and Ir dispersion (electrochemical Ir surface area, cm_Ir_^2^ g_Ir_^–1^) at constant cell current density, j (A cm_geo_^–2^). Hyperbolic curves indicate lines of constant specific catalytic activity, j_0_ (A cm_Ir_^–2^). Reduced L_Ir,geo_ at lower (case 1) or comparable (case 2) Ir dispersion requires intrinsically more active electrocatalysts. Reducing L_Ir,geo_ at comparable j_0_ requires a higher Ir dispersion (case 3).

Water electrolysis for the production of green hydrogen, driven by renewable energy, is an important Power-to-Gas technology shaping our future energy infrastructure.^1,2^ Low-temperature Proton Exchange Membrane Water Electrolyzers (PEM WE) offer a number of advantages over the more established liquid alkaline electrolyzer technology. However, the harsh corrosive acidic conditions in PEM electrolyzers necessitate the utilization of noble metal-based catalysts at cathode and anode.^3−5^ More specifically, rutile IrO_2_ is the current state-of-the-art catalyst for the anodic *oxygen evolution reaction* (OER).^6,7^ There is currently no viable alternative to Ir-based anode catalysts, despite its limited reserves, high cost, and limited current recyclability.^8,9^ Future PEM water electrolyzer technologies will have to lower their power-specific iridium demand (g_Ir_ kW^–1^) by a double-digit factor^10^ at comparable or improved cell voltages (voltage efficiencies) to enable scalability. To address this challenge, the geometric Ir loading of PEM water electrolyzer anodes will have to drop, while maintaining a comparable anode catalyst layer thickness.^11^ This requires the development of new electrocatalysts with reduced Ir packing density (g_Ir_ cm^–3^).^12−14^Figure 1 illustrates the relevant design constraints schematically. Desired design points at lower geometric Ir loadings, L_Ir,geo_ (g_Ir_ cm^–2^) (dashed iso-Loading hyperbolic lines in Figure 1a), become accessible through lower packing densities at constant catalyst layer thickness (arrow). Lower packing density means reduced Ir volume fractions of the catalyst material. This has immediate impact on the required catalyst reactivity and Ir dispersion if the hydrogen productivity and cell current, j, are to be maintained in the lower-loaded anode. Figure 1b illustrates that if the atomic Ir dispersion (specific electrochemical Ir surface area, ECSA_Ir)_ does not increase at lower Ir loading (cases 1 and 2 in Figure 1b), then the intrinsic OER catalytic activity, j_0_ (see hyperbolic isoactivity lines), must rise. Figure 1 describes both unsupported bulk and supported catalysts, but the presence of the support offers the possibility of lowering the Ir packing density without changing the quantitative chemical composition of the Ir-based catalytic active phase.

Viable OER electrocatalysts for acidic environments must exhibit sufficient electrical conductivity, high catalytic OER reactivity^15,16^ and sustained chemical and structural stability over prolonged operation times.^17,18^ Today, Ir or IrO_*x*_ particles dispersed on TiO_2_ powder supports are one of the most promising and versatile catalyst–support materials candidate to meet the physicochemical and catalytic requirements of PEM WE anodes.^11−14,19^ The reason for this is twofold: First, the chemical state of the catalytic active IrO_*x*_ component can range from (i) Ir-derived hydrous IrO_*x*_ (often referred to as IrO(OH)_*x*_) with its unique metal–ligand covalency,^7,20^ its high coverage of electrophilic oxygen ligands coupled to high catalytic oxygen evolution activity^21^ all the way to (ii) crystalline, rutile IrO_2_ particles with their unique structural and chemical stability and high electrical conductivity.^12,13,22^ This uniquely wide bandwidth of structural and electronic states of Ir offers a balanced design of the activity, conductivity, and stability of the catalytic active phase.

Second, the TiO_2_ support offers excellent electrochemical corrosion stability and isomorphic rutile surface facets, although at very low electrical conductivity. This is why the discussion of the IrO_*x*_/TiO_2_ catalyst–support interfaces requires careful consideration of the support material properties. Among the prevalent TiO_2_ allotropes, rutile demonstrates remarkable stability,^23^ whereas anatase exhibits the lowest surface enthalpy,^24^ enabling stabilization at smaller particle sizes and higher surface areas. Crystalline anatase TiO_2_ (henceforth refer to as a.TiO_2_) also possesses the highest electrical conductivity.^25^ Still, when dispersing Ir or IrO_*x*_ nanoparticles (NPs) on the surface of a.TiO_2_, electrical currents flow preferentially across the supported IrO_*x*_ layer rather than across the support material. This is why the IrO_*x*_ NPs serve as both catalytically active sites and the major conductive components of the supported catalyst.

A key performance parameter of low-Ir-content Ir/TiO_2_ or IrO_*x*_/TiO_2_ interfaces is their chemical and structural stability under operating conditions. This requires the use of *operando* or online stability measurements. Past stability analyses of IrO_*x*_/TiO_2_ catalysts considered principal factors such as backing electrode corrosion,^17,26−28^ operational stability, and material stability.^16^ More specifically, catalyst dissolution,^29^ support passivation,^30^ bubble formation,^31^ surface blocking, and changes in particle size distribution (PSD) were also found to affect the overall stability.^8,32^ Mechanistically, Kasian et al. reported a dominant high-potential dissolution route, leading to the formation of an unstable IrO_3_ intermediate. The IrO_3_ decomposes into IrO_2_ and O_2_ or transforms into the soluble anion IrO_4_^2–^.^33^ Numerous experimental protocols have been established for assessing OER catalyst stability, including determining catalytic lifetime under constant applied voltage/current^34−37^ or evaluating performance loss after a specified duty-cycle protocol^38^ or based on the so-called S-number.^18,39^ Thus, the IrO_*x*_/TiO_2_ catalysts are promising electrocatalytic materials, which are still not properly understood. Characterizing IrO_*x*_/TiO_2_ systems involves considering their structure, morphology, activity, stability, and electrical conductivity.^12,13^

The present study explores materials design trade-offs in the IrO_*x*_/a.TiO_2_ catalyst–support system with respect to the OER activity, particle size, Ir packing density, Ir atomic stability, Ir weight loading, and electrical conductivity. In doing so, we identify a reduced-Ir-content OER catalyst, Ir@IrO(OH)_*x*_/a.TiO_2_, with sufficiently high OER Ir mass activity to offset performance losses in low Ir-loaded catalyst layers. Atomic-level high-angle annular dark field (HAADF) and energy dispersive X-ray (EDX) microscopy and *operando* X-ray spectroscopy (XAS) will provide insight in the origin of the high Ir mass activity and will serve to link Ir atom dissolution to the emergence of catalytic active electrophilic Ir–O^I–^ ligands.

## 2 Experimental Section

### 2.1 Synthetic Protocol

Ir NPs supported on anatase TiO_2_ NPs (Ir/a.TiO_2_) with different Ir loading levels, x weight (wt)% (x = 30, 40, 50, 60, 70, and 80), were synthesized using the following protocol. Approximately 1.22 g of dihydrogen hexachloroiridate(IV) hydrate (H_2_IrCl_6_·6H_2_O, Ir content: 36–44%, Sigma-Aldrich) was dissolved in 1 L of water to create a precursor stock solution. The concentration of Ir^4+^ was redetermined using ICP-OES. Subsequently, 100 mL of the precursor solution were mixed with the corresponding amount of a.TiO_2_ dispersed in isopropanol (IPA) using sonication. 0.1 M KOH solution was gradually added under stirring and heating to 80 °C to initiate hydrolysis, which was maintained for 1 h. After adjusting the pH of the resulting solution to approximately 8, the mixture was stirred for an additional 30 min to deposit metallic Ir NPs on the support surface. The resulting black powder was obtained by washing, centrifuging, and drying. The catalysts were thermally treated in synthetic air at 300 °C for 1 h. Additional details of the synthetic protocols are provided in the Supporting Information (SI), and an illustration of the suggested synthetic reaction mechanism is depicted in Figure S1 and further discussed in Supplemental Note 1.

### 2.2 Physico-chemical Characterization

*X-ray diffraction (XRD) profiles* were obtained using a D8 Advance X-ray diffractometer (Bruker AXS) equipped with a Cu Kα source and a variable divergence slit. Automatic sample selection was facilitated by a position-sensitive detector. Data were collected in the 2θ range of 10° to 95°, with an angle increment of 0.05° and a measuring time of 6 s per step, without sample rotation. The XRD profiles were background-subtracted and normalized to the highest reflection of anatase TiO_2_, which served as the support material. The full width at half-maximum (fwhm) of Bragg reflexes, corrected for instrumental line broadening, was used to determine the crystallite size of the support and the Ir phase. This was achieved by employing the Scherrer equation with a shape factor constant, κ, of 0.89.

*Elemental analysis via inductively coupled plasma optical emission spectroscopy (ICP-OES)* was performed by using a 715-ES-ICP analysis system (Varian). The Ir standard concentrations ranging from 0.5 to 6.0 ppm were prepared, and the Ir concentration was determined at several selected wavelengths, including 212.681, 224.268, 236.804, 254.397, and 263.971 nm. Detailed sample preparation procedures can be found in the SI.

*Elemental analysis using X-ray fluorescence (XRF)* was conducted using a Bruker AXS S8 TIGER High-Performance Wavelength Dispersive XRF spectrometer, employing the QUANT-EXPRESS calibration. Approximately 20 mg of each sample was mounted in 5 mm XRF open-ended aperture cups (Chemplex Industries) covered with a 2.8 μm thick Prolene film. The measurement was performed three times to obtain an error margin. As noted in Supplemental Note 2, XRF elemental analysis provides the most reliable composition information; thus, all catalytic inks’ preparation in this study are normalized to the Ir-content, determined via XRF.

*Elemental analysis using transmission electron microscopy (TEM) and energy-dispersive X-ray (EDX)* was carried out using an FEI TECNAI G2 20 S-TWIN TEM equipped with a LaB6 cathode and a GATAN MS794 P CCD camera (ZELMI). The microscope operated at an acceleration voltage of 200 kV. The as-prepared catalyst powders were suspended in ethanol using horn-sonification in a 6 mL glass vial, and then 10 μL of the suspension was pipetted onto a carbon-coated copper grid (400 mesh, Plano) and dried for 15 min at 70 °C. TEM-EDX analysis was performed using an r-TEM SUTW detector (EDAX Inc., NJ, USA) with a Si(Li) detector, achieving an energy resolution lower than 136 eV for Mn Kα, and detecting elements from Boron (Z = 5). The size distributions of iridium and the support were determined by measuring around 500 particles using ImageJ 1.53t software (the U.S. National Institutes of Health).

*High-resolution scanning transmission electron microscopy (HR-STEM)* was conducted using a Jeol JEM-ARM300F2 electron microscope operated at a 300 kV accelerating voltage. The JEM-ARM300F2 is a probe aberration-corrected STEM/TEM instrument equipped with a cold field emission electron (cold FEG) source and a JEOL ETA Probe Cs corrector. For high-angle annular dark-field (HAADF) imaging, camera lengths of 8 and 10 cm were used, corresponding to 68–280 mrad and 54–220 mrad. EDX maps were recorded using a windowless dual SDD detector system with dimensions of 2 × 160 mm^2^ solid angle of 2.2 sr. The sample was dispersed in milli-Q water and drop-deposited onto lacey carbon-coated copper grids.

*Conductivity measurement*. The electrical resistivity of the IrO_*x*_/a.TiO_2_ system was determined by using a commercial potentiostat connected to a custom-built powder conductivity apparatus consisting of upper and lower pistons, as depicted in Figure S2. For high conductivity powders, Potentiostatic Electrochemical Impedance Spectroscopy (PEIS) was conducted using the Gamry Preference 3000 Potentiostat/Galvanostat/ZRA. The frequency range used was from 10^6^ to 1 Hz, with 10 reading points per decade. The electrical resistance (R) between the upper and lower brass pistons was obtained by fitting the data using the Gamry Echem Analyst software. In the case of low conductivity powders, the Keithley 6430 Sub-Femtoamp Remote SourceMeter was employed to measure high resistivity, at a voltage of 1.0 V. The thickness of the compressed powder inside the cylinder was measured using a manual high-precision caliper. The inner diameter of the cylinder was 0.8 mm. A slight pressure of 1.5 MPa, which includes the weight of the top part (approximately 0.770 MPa), was applied during this process. The initial thickness, denoted as h_0_, was subsequently measured to assess the *packing density of the powder*. Additional information can be found in Table S1 and Supplemental Notes 3 and 5 within the SI.

*Inductively coupled plasma mass spectrometry (ICP-MS) coupling scanning flow cell (SFC)*. The quantification of dissolved metal ions was carried out in argon-purged 0.05 M H_2_SO_4_ using an SFC coupled to an ICP-MS instrument. The detection limit for Ir ranged from 0.1 to 1 ppt. A graphite rod served as the counter electrode, and a Ag/AgCl electrode (Metrohm) was used as the reference electrode. The concentrated sulfuric acid (H_2_SO_4_ 98%, Merck) was diluted in ultrapure water (PureLab Plus system, Elga, 18 MΩ cm, TOC < 3 ppb) to prepare the electrolyte. The flow rate through the cell was maintained at 352 μL min^–1^. The stability and accuracy of the ICP-MS instrument (NexION 300X, PerkinElmer) were ensured through daily calibration and the addition of an internal standard solution downstream from the flow cell. The isotopes ^187^Re and ^45^Sc were used as measured isotopes for calibration purposes. Electrochemical measurements using both setups were performed using a Biologic potentiostat VSP-150.^40^

*Operando XAS experiments* have been performed at beamline P64 of Petra III at DESY (Hamburg, Germany). Measurements were performed at the Ir L_3_-edge. A Si(111) double-crystal monochromator was used for energy selection, with a PIPS detector used to collect the XAS signal in fluorescence mode. The electrochemical cell was described elsewhere.^41^

## 3 Results and Discussion

### 3.1 Synthesis and Physico-chemical Characterization of TiO_2_ Supported Ir-Based Catalysts

A series of TiO_2_-supported hydrous Ir@IrO(OH)_*x*_ core–shell nanoparticle catalyst–support materials were synthesized using a liquid precursor-based precipitation technique. The notation Ir@IrO(OH)_*x*_ refers to a metallic Ir particle core with a hydrous Ir oxide shell on its surface, as the experiments below will show. As the as-prepared TiO_2_-supported NPs showed bulk metallic Ir properties prior to catalytic activation and testing in liquid electrolytes, we refer to the synthesized precatalyst materials as “x% Ir/a.TiO_2_” samples, where x indicates the iridium weight loading. We are aware that the Ir/a.TiO_2_ samples are merely precatalysts that convert into their catalytic active core@shell Ir@IrO(OH)_*x*_/a.TiO_2_ form under electrochemical conditioning.

**Figure 2:**
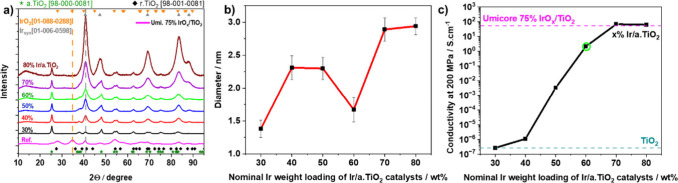
Physico-chemical characterization of Ir nanoparticles supported on a.TiO_2_ powders. (a) XRD reflection patterns, specific reflection for the plane (101) of tetragonal IrO_2_ and specific reflection for the plane (111) of cubic Ir are indicated by orange and dark gray vertical dash lines, respectively. Various Ir% weight loadings and the commercial catalyst are color-coded in 10% weight intervals ranging from black for 30% Ir/a.TiO_2_ to dark red for 80% Ir/a.TiO_2_, dark cyan for the TiO_2_ support, and magenta for the Umicore catalyst. Reference reflection patterns from the powder diffraction file (PDF) are provided for comparison (pdf number given in parentheses). (b) Average particle size from TEM particle size histograms. (c) Electrical conductivity of the anatase TiO_2_ support (dark cyan dashed horizontal line), the synthesized Ir/a.TiO_2_ catalysts, and the commercial reference (pink dashed horizontal line).

The as-prepared Ir/a.TiO_2_ precatalyst samples were characterized ex-situ with respect to their crystalline phase structure, their TEM-based particle size, their chemical composition, and electrical powder conductivity. The summarized results are presented in Figure 2, with more details in Figures S4 and S5 and Table S2.

Accurate analysis of the Ir weight loadings of the supported catalysts (nominally ranging from 30 to 80 wt %) was carried out using ICP-OES, EDX, and XRF. Figure S3 illustrates the correlations of actual and nominal loadings. We found XRF analysis to be the most reliable technique, least affected by rutile IrO_2_ and TiO_2_ solubility, and used it for Ir mass normalizations in all electrochemical tests.

The XRD patterns of the Ir/a.TiO_2_ catalysts are presented in Figure 2a. The observed reflections at 2θ values of 34.656° and 40.669° correspond to the crystallographic planes (101) of tetragonal IrO_2_ and (111) of face-centered cubic (fcc) metallic Ir, respectively. The XRD results indicate the presence of metallic iridium phases in the pristine catalysts, however, no crystalline IrO_2_ phases were detected. Amorphous short-range ordered IrO_*x*_ on the surface of the Ir NPs cannot be excluded with certainty based on the patterns. The peak area at 2θ of 40.669° increased monotonically with higher Ir loadings, with the 60% Ir/a.TiO_2_ exhibiting a slightly broadened peak. The composition of the catalysts and the presence of metallic iridium support the role of isopropanol as a dispersing medium for a.TiO_2_ and a reducing agent for iridium precursors (see Figure S1). In contrast, iridium in the reference catalyst is present in both crystalline IrO_2_ phases and metallic Ir phase. Crystalline (domain) sizes of all catalyst materials are listed and compared to particle size in Table S2 and Supplementary Note 6.

The morphology and particle size of the Ir/a.TiO_2_ samples were investigated by using TEM. The Ir-based mean particle size on the support surface increased from 1.4 to 3.0 nm (Figure 2b) with increasing Ir content. The 60% Ir/a.TiO_2_ sample proved to be a consistent outlier with a reduced particle size anomaly of 1.67 nm, only slightly larger than that of the 30% Ir/a.TiO_2_ sample of 1.38 nm. This suggested a saturated Ir particle loading level, where the precipitation and distribution of smaller Ir NPs directly onto the support surface becomes more favorable compared to lower Ir loadings. By contrast, at loadings in excess of 60 wt % the deposition of Ir NPs occurs on both free and Ir-covered regions of the support surface, leading to agglomeration and increased particle size (∼3.0 nm).

Another important material property of a viable Ir/a.TiO_2_ electrocatalyst is its electrical conductivity. Electrical conductivities of the catalysts and reference powders were measured using a two-point probe method (Figures S2 and S15) at 200 MPa pressures, with results presented in Figure 2c. A sharp conductivity increase over 7 orders of magnitude (0.268 μS cm^–1^ to 2.139 S cm^–1^) accompanies the increase in iridium loading from 30 to 60 wt %. Notably, the conductivity of the supported catalyst materials increases by a factor of 2 ·10^6^ between just 40 and 60 wt % of Ir. The conductivity continued to increase further and plateaued at and beyond 70 wt % with a value of 42 S cm^–1^ matching the conductivity of the Elyst reference catalyst (red dashed line in Figure 2c). More details on the physicochemical properties of the as-prepared Ir/a.TiO_2_ samples, in particular Ir packing density values, are given in Table S1, Figures S4 and S5.

### 3.2 Electrocatalytic Performance of Supported Ir@IrO(OH)_*x*_ Particle Catalysts

**Figure 3:**
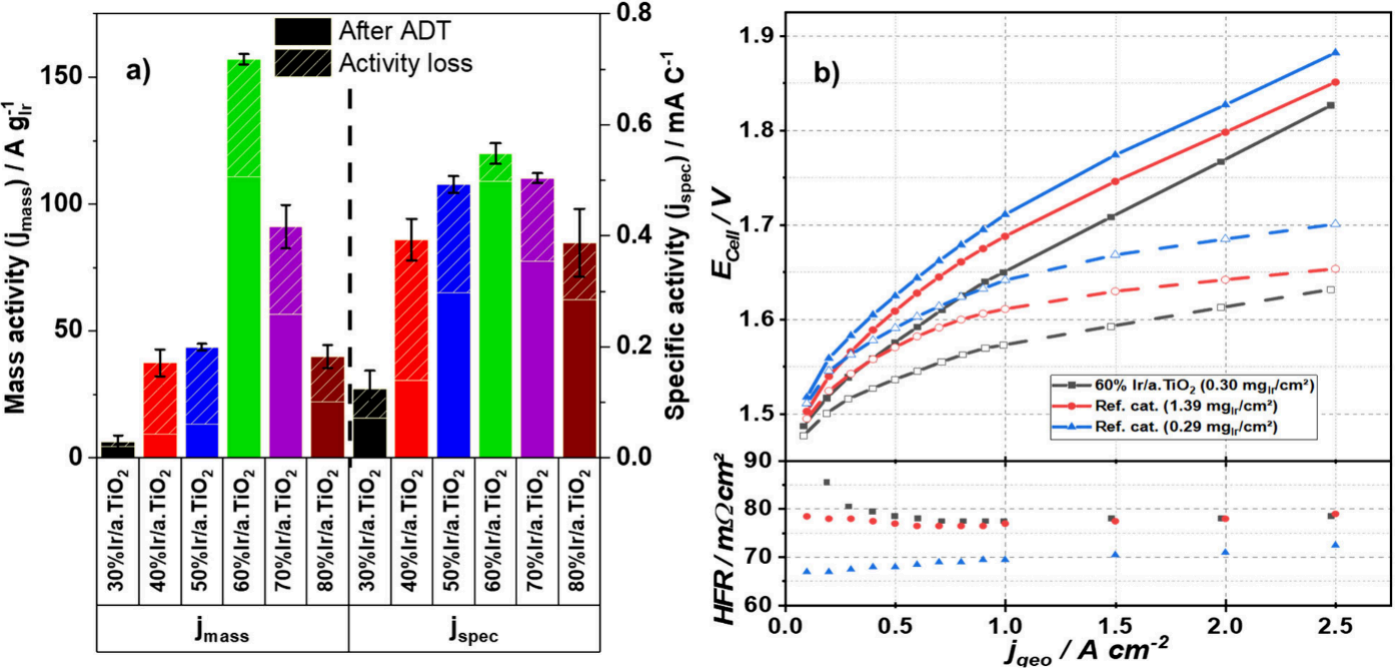
Electrocatalytic OER activities of activated Ir/a.TiO_2_ catalysts. (a) Rotating disk electrode (RDE) activity and activity losses of activated Ir/a.TiO_2_ catalysts before and after degradation tests. The OER activities after activation (AA), the activity loss (hatched portion) during accelerated degradation tests (ADT), and the remaining activity post-ADT are shown. ADTs consisted of 5000 voltammetric pulses between 1.23 and 1.6 V. The OER currents at an overpotential of 300 mV were normalized to the total Ir loading on the electrode, referred to as mass activity (j_mass_, A g_Ir_^–1^) or to the anodic interfacial capacitive charge q*, presented as specific activity (j_spec_, A C^1–^). (b) Single cell MEA polarization tests of the 60% Ir/a.TiO_2_ anode catalyst compared to a commercial IrO_2_/TiO_2_ anode catalyst (Umicore Elyst); 60% Ir/a.TiO_2_ (0.30 mg_Ir_ cm^–2^ black solid and dashed line), reference (1.39 mg_Ir_ cm^–2^ red solid and dashed line; 0.29 mg_Ir_ cm^–2^ blue solid and dashed line). High Frequency Resistance (HFR)-corrected values are shown as hollow symbols and dashed lines. MEA specifications: Nafion NR 212 membrane, 5 cm^2^, decal transfer process; cathode catalyst loading of ∼0.11 mgPt cm^–2^. RDE measurement conditions: Au substrate for the working electrode, 0.05 M H_2_SO_4_, 1600 rpm. The loading of Ir was approximately 20 μg _Ir_ cm^–2^.

Upon exposure to the electrolyte, the metallic Ir NPs form hydrous Ir oxides, termed IrO(OH)_*x*_, on their surface, resulting in Ir@IrO(OH)_*x*_ core–shell particles. However, for the sake of notational simplicity, we will continue to refer to the catalyst samples as Ir/a.TiO_2_. Figure 2c and Figure 3a evidence the relation between electrical conductivity and the accessibility of active sites, i.e., the catalytic activity. Samples with low electrical conductivity and Ir content (≤50 wt %) exhibit low Ir mass-normalized OER current density, referred to as Ir mass activity, and suffer from substantial performance losses (e.g., 80% loss for the 40% Ir/a.TiO_2_, see hatched regions of bars in Figure 3a) after the accelerated degradation test (ADT). As the Ir loading increased from 50 to 60 wt %, the mass activity sharply improved from 43 mA g_Ir_^–1^ to about 150 mA g_Ir_^–1^, more than tripling in value. At higher loading contents (≥70 wt %), where the conductivities plateaued (Figures 2c), agglomeration observed in the TEM images (Figures S4) lead to an increase in the mean Ir particle size (Figures 2b) and a decrease in dispersion and surface sites, resulting in a lower Ir mass activity. After the ADT, the mass activity of the 60% Ir/a.TiO_2_ catalyst decreased by 29%, reaching 110 mA g_Ir_^–1^. However, the j_spec_ of 60% Ir/a.TiO_2_ decreased by only 9% and remained significantly higher than the other samples. Since the specific activity reflects the average performance of each active site, the Ir@IrO(OH)_*x*_ core–shell particles appeared to experience a significant stabilization on the TiO_2_ support surface. This may suggest the contribution of catalyst–support interactions (CSI) in the Ir/a.TiO_2_ system. The favorable OER activity of the 60 wt % sample was further confirmed by comparison with commercial references, as shown in Figure S6c-d. The commercial state-of-art Umicore Elyst catalyst demonstrated a 7 times lower mass activity and a 3 times lower specific activity before the ADT. After the ADT, the reference catalyst experienced a 25% loss in mass activity and a 32% loss in specific activity, widening the net performance benefit of the 60 wt % Ir/a.TiO_2_ sample.

To assess the catalytic performance of the 60%Ir/a.TiO_2_ catalyst versus the Umicore Elyst 75% IrO_2_/TiO_2_under more realistic conditions, PEM single-cell electrolyzer measurements were conducted. Membrane electrode assemblies (MEAs) of the catalysts were prepared using a decal process. The catalyst layers were first spray-coated (anode side) and bar-coated (cathode side) onto polymer decal foils and subsequently transferred onto the Nafion membrane using a hot pressing decal transfer process. As shown in Figure 3b, the Umicore reference MEA with higher iridium loading (1.39 mg_Ir_ cm^–2^) outperformed that with lower loading (0.29 mg_Ir_ cm^–2^). The thicker anode layer of the higher Ir loading contributed to the larger High Frequency Resistance (HFR) value. On the other hand the 60%Ir/a.TiO2 catalyst exhibited significantly lower kinetic overpotentials compared to either reference test across the current load range. Its HFR value is comparable to that of the high-loaded reference, which is consistent with a thicker anode layer caused by its lower Ir packing dendsity. Mass-normalized currents (Figure S7a) revealed a ***36.4-fold*** higher OER mass activity (4004 A g_Ir_^–1^) for 60% Ir/a.TiO_2_ compared to the Umicore Elyst 75% IrO_2_/TiO_2_ reference (110 A g_Ir_^–1^) at a cell voltage of 1.53 V. As a result, the specific iridium demand, at 70% lower heating value (LHV) based voltage efficiency (Figure S7b), was reduced to 0.075 g_Ir_ kW^–1^, which is 5–6 times lower than a literature benchmark^12^ and approaching the target of 0.01 g_Ir_ kW^–1^.^9^

### 3.3 In-Situ Metal Dissolution Studies and the Stability of Ir/a.TiO_2_ Catalysts

To examine the impact of Ir and Ti dissolution on the performance stability of the Ir/TiO_2_ catalysts, we employed a scanning flow cell technique coupled to ICP-MS.^5,29,39^ This technique investigates in situ metal dissolution rates, dissolution amounts, and concomitant OER activities. Three samples, 50% Ir/a.TiO_2_, 60% Ir/a.TiO_2_, and 70% Ir/a.TiO_2_, as well as the commercial reference catalysts were investigated.

**Figure 4:**
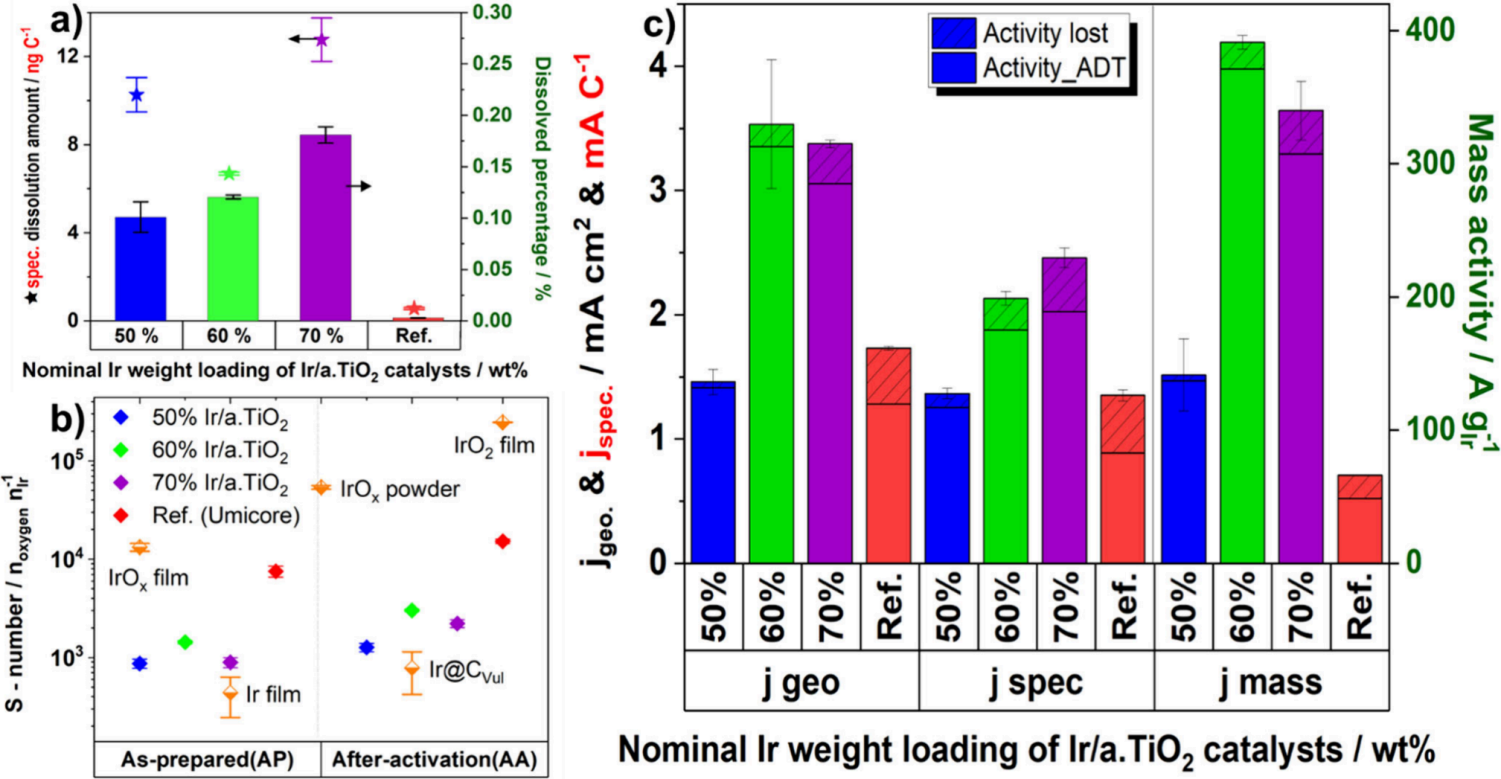
In-situ Ir dissolution and OER performances of 50%, 60%, and 70% Ir/a.TiO_2_, and Umicore reference were averaged from three consecutive short ADTs, measured using ICP – MS coupled SFC. (a) Ir- dissolution amount normalized by anodic charge (d_a.spec._, ng_Ir_ C^–1^), presented as scatter plot on left *y*-axis, and Ir-dissolved percentage normalized to the total Ir loading amount on the electrode (%), displayed as column plot on the right *y*-axis. (b) Stability number, defined as the ratio between the amount of produced oxygen, calculated from total charge and the dissolved iridium (S-Number, n_oxygen_ n_Ir_^–1^), obtained from a low potential sweep (5 mV s^–1^), ranging from 0 to 1.55 V_RHE_, for both as-prepared and after activation samples. The corresponding data for the reference materials (depicted as half-down orange diamond scatters) were taken from the literature.^42^ (c) Catalytic activity represented as the current normalized by the total anodic charge q* (j_.spec_, mA C^–1^) or normalized by the surface area (j_geo._, mA cm^–2^) (left *y*-axis), or normalized by the Ir loading on the electrode (j_mass._, A g_Ir_^–1^) (right *y*-axis). A solid diagonal-up pattern indicates the activity loss after ADT, compared to the highest activity state. Measurement conditions: Au working electrode (WE), 0.05 M H_2_SO_4_. For the synthesized catalysts, the loading of Ir was approximately 10 μg cm^–2^, and for the commercial reference catalyst, Ir loading was increased to 25 μg cm^–2^ to achieve a better distribution of the catalyst spots on the WE.

After each ADT, the integrated geometric iridium mass dissolution of the 60% Ir/a.TiO_2_ material (Figure 4a) was similar to 50% Ir/a.TiO_2_, and about two-thirds of 70% Ir/a.TiO_2_. The dissolved Ir amounts of 60% Ir/a.TiO_2_ were thereby, as expected for Ir@IrO(OH)_*x*_, larger than that of the crystalline IrO_2_ reference catalyst, more specifically, 0.1% for 60% Ir/a.TiO_2_ and 0.002% for the reference. Low dissolution of the reference catalyst is related to its very large crystalline IrO_2_ particles, coupled to their much higher Ir weight loading, as shown in Figures S4 and S12. Besides, the dissolved amount of Ti after ADT was negligible for all investigated catalysts (Figure S8).

To further evaluate the corrosion stability, the well-documented S-numbers were evaluated (Figure 4b). Despite small particle sizes (approximately 1–3 nm), the S-numbers of all synthesized Ir/TiO_2_ catalysts were roughly 3-fold larger than that of a metallic Ir film. Particularly noteworthy is the comparison with the Ir film, where the S-number of the 60 wt % Ir deposited on TiO_2_ shows a significant increase after activation and stabilization during ADT. This is additional evidence for the presence of catalyst–support-interactions (CSI) that chemically stabilize Ir@IrO(OH)_*x*_ NPs on the anatase TiO_2_ support. After the ADT, the S-number of 60% Ir/a.TiO_2_ is ∼4-fold lower than the reference and a hydrous IrO_*x*_ thin-film and ∼14-fold lower than a hydrous IrO_*x*_ powder reported in literature.^18^ Importantly, the S-number of the 60% Ir/a.TiO_2_ sample was ca. 2-fold larger than that of all other Ir/a.TiO_2_ samples, suggesting that interactions between the anomalously small Ir particles and their homogeneous spatial distribution (see TEM below) helped prevent Ir dissolution.

Catalysts with higher S-numbers are expected to show a lower mass activity loss after ADT. However, Figure 4c shows a different trend. While the OER mass-based activity of 60% Ir/a.TiO_2_ catalyst decreased by a mere 5% after ADTs, the commercial reference catalyst revealed a significant 26% activity loss in the online ICP-MS experiments. In other words, despite the 50× higher relative Ir-dissolution of the 60% Ir/a.TiO_2_ (Figure 4a), it showed an unexpected 5× lower mass activity loss. The answer lies, in part, in the specific OER activities. Initially, the optimally synthesized catalyst demonstrates a 1.5× higher specific activity than the reference, yet after the ADT, this difference increased to 2.1×. Thus, the activity of Ir-active sites in the commercial catalyst decays faster compared with those of the 60% Ir/a.TiO_2_ sample.

Our SFC-ICP-MS study revealed that during ADTs, the Ir mass dissolution does not appear to be necessarily the primary factor controlling the loss of the OER activity (Figure 4a and 4c). Rather, the loss of intrinsic specific activity of surface Ir sites, likely related to changes in the chemical nature of the active sites, governs the resulting mass activity degradation. Therefore, it is imperative to complement electrochemical and online ICP-MS techniques with advanced characterization methods for understanding the degradation mechanisms of active sites at the atomic scale, presented in the following sections.

### 3.4 Microstructural Insight in the Stability of the 60% Ir/a.TiO_2_ Catalyst

Temporal trajectories in the particle size distribution (PSD) of metallic NPs on support materials can be mechanistically associated with coalescence, dissolution/redeposition, and Oswald ripening.^16^ However, the physical contact between the surface sites and the support can, in certain cases, decrease the dissolution rate, hinder particle movement, and maintain a homogeneous distribution of surface sites during ADT.^8^ In our efforts to better understand the influence of the Ir particle morphology and composition on the catalytic stability, we now focus on high resolution scanning transmission electron microscopy (HR-STEM) combined with EDX mappings of our most promising 60% Ir/a.TiO_2_ catalyst. We investigated this sample in different chemical states: pristine as-prepared (60% Ir/a.TiO_2__AP), after activation (60% Ir/a.TiO_2__AA), and after the stability test (60% Ir/a.TiO_2__ADT).

**Figure 5:**
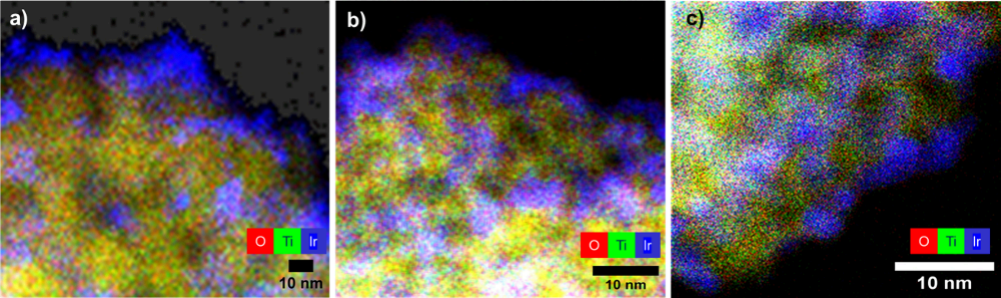
Overlayed EDX elemental mappings of O (red), Ti (green), and Ir (blue) of the 60% Ir/a.TiO_2_ catalyst, at three different states: (a) the as-prepared state (AP); (b) after electrochemical activation and oxidation (AA), and (c) after the accelerated degradation test (ADT). Combination of red and green makes yellow color of TiO_2_.

In the as-prepared (AP) state, HR-STEM/EDX (Figures 5a and S9) revealed that the Ir NPs of the 60% Ir/a.TiO_2_ catalyst and its active surface sites are uniformly distributed across the support surface, unlike the commercial reference catalyst with its very large Ir aggregates (Figures S4, S12). EDX elemental mappings of Ir, O, and Ti are in agreement with the presence of metallic iridium, with oxygen being mainly located at titanium, consistent with the specific reflections on XRD patterns. Notably, Figure S9g demonstrates the presence of small clusters and individual iridium atoms on the a.TiO_2_ surface. The Ir–average particle size on the a.TiO_2_ support surface is clearly observable in Figure 5a and Figure S9e-g. During the activation step, the metallic Ir NPs undergo an electrochemical oxidation of their surface into a hydrous IrO(OH)_*x*_ (magenta point regions in Figure 5b). There is negligible alteration in their distribution or particle size due to the short duration of this step. Therefore, in the upcoming discussion, the focus on the AA state is not on the morphology of the Ir NPs, but rather on the formation and variation of the iridium (hydr-)oxide layer on the outermost surface and its influence on the stability of the OER catalyst.^33^

Images of the electrocatalytically tested 60% Ir/a.TiO_2__ADT (Figures 5c and S11) did not show any presence of enlarged and rounded shapes or isolated Ir NPs, which could be formed through coalescence or Ostwald ripening of smaller NPs. This evidenced an unexpected morphological stability of the IrO(OH)_*x*_ NPs on the TiO_2_ support surface following the decrease in mass-based activity as well as the dissolution resistance, and corroborates the role of the TiO_2_ support in stabilizing the catalyst PSD.

For an overall comparison of the changes in the catalyst’s morphology, PSD, composition, and the Å-sized Ir oxide overlayers following the ADT, HR-STEM images of 60% Ir/a.TiO_2_, provided in Figure 5 and Figures S9, S11, can be put together with the commercial reference catalyst, provided in Figure S12. Figure S13 further demonstrates how the crystalline Ir surface structure of the pristine NPs transforms into an indistinct amorphous appearance after the ADT stability test. Furthermore, the absence of changes in the Ir PSD after ADT (Figure S11) suggests negligible iridium dissolution.

**Figure 6:**
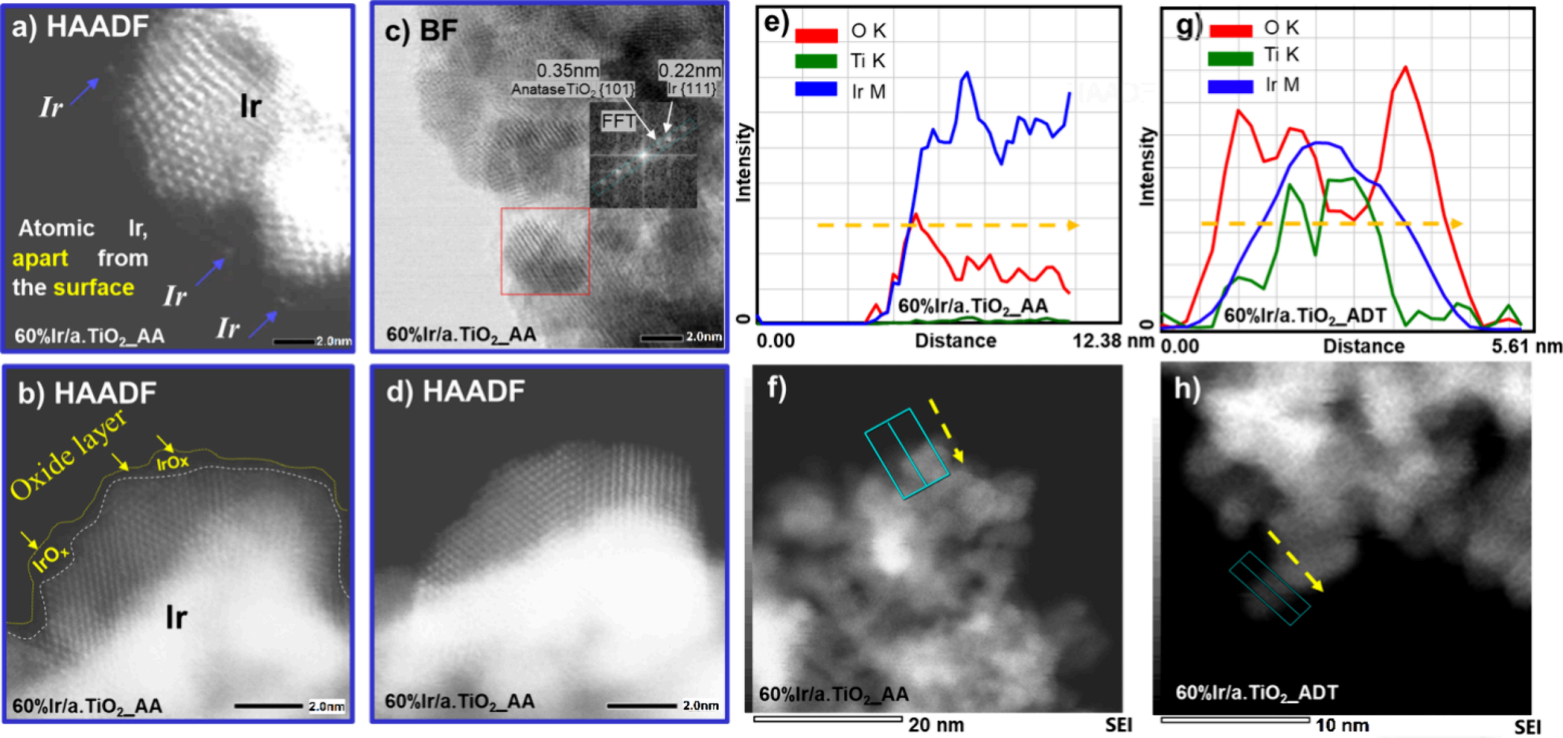
HR-STEM images and EDX line scans of 60 wt % Ir/a.TiO_2_ after catalytic activation. (a and b) HAADF images of thin hydrous IrO_*x*_/IrO(OH)_*x*_ layers formed after catalyst activation on the surface of TiO_2_-supported Ir NPs as observed before EDX mapping. (c) BF image of supported catalyst after activation with a Fast Fourier Transform inset, revealing the metallic crystalline bulk structure of Ir NPs on the crystalline a.TiO_2_ support. (d) HAADF image after a rapid EDX mapping scan at 300 kV for 2 min, where the electron beam has caused degradation of the hydrous oxide layer. (e) EDX line scan of Ir 60 wt % on TiO_2_ after catalytic activation displaying intensity profiles of O, Ti, and Ir corresponding to the rectangular area indicated in (f). (f) HAADF images of Ir 60 wt % on TiO_2_ after catalytic activation. (g) EDX line scan of Ir 60 wt % on TiO_2_ after stability ADT illustrating intensity profiles of O, Ti, and Ir corresponding to the rectangular area indicated in (h); (h) HAADF images of Ir 60 wt % on TiO_2_ after stability ADT. Color code: oxygen (green), titanium (blue), and iridium (red).

To learn more about the formation of amorphous hydrous IrO(OH)_*x*_ on Ir NPs after catalyst activation, Figures 6a and 6b present atomically resolved HAADF images: A thin hydrous IrO(OH)_*x*_ layer is observed on the outermost surface of the crystalline iridium NPs, less than 1 nm in thickness (Figure 6b). This layer is identified as an oxygen-rich layer based on quantification from the counts-map and HR-STEM detection. This layer also supports individual single iridium atoms clearly separated from the crystalline iridium nanoparticle surface (Figure 6a). The oxide layer is visible only when combining bright field and dark field images, as represented by the dotted lines in Figures 6b, S14c. Note that the preparation of HR-STEM samples involved high energy sonication to extract the electrochemically treated catalyst ink from the working electrode surface. This is why the observed oxide layer must exhibit considerable mechanical stability on the particles. The hydrous oxide layer behaved chemically unstable under prolonged electron beam irradiation during EDX-mappings (Figure S14b, d). As the hydrous oxide layer diminished, the surface of iridium nanoparticles (NPs) directly beneath this layer appeared to transform from crystalline structures to more amorphous structures, as illustrated in Figure 6d. In Figure 6c, the inset displays fast Fourier transform (FFT) analysis. The FFT analysis revealed an interplanar distance of 0.35 nm, corresponding to the anatase TiO_2_ (101) plane, and an interplanar distance of 0.22 nm, corresponding to the Ir (111) plane. This confirms that, in the bulk of the larger particles, iridium predominantly exists in its metallic form, with the oxide layer only present on the outermost surface of the catalysts.

Finally, to learn more about the structural and chemical state of the 60% Ir/a.TiO_2_ sample after catalytic testing, cross-sectional EDX of the 60% Ir/a.TiO_2__AA and 60% Ir/a.TiO_2__ADT samples were analyzed (Figure 6e-h). The AA sample revealed a very thin oxidic shell on a primarily metallic Ir particle core with the O K-edge intensity peaking around 1.5 nm below the surface (Figure 6e). After the ADT (Figure 6g and 6h), the O K-edge intensity significantly increased in the outermost layers with two radially symmetric intensity maxima, a clear indication for an O-rich IrO_*x*_ particle shell. Ir M- and Ti K-edge intensities, by contrast, peak at the particle center suggesting metallic core-oxide shell structures. The composition of the oxygen layer after the ADT is electrochemically unstable and cannot be analyzed under electron beams.

The HR-STEM images and EDX results provide direct visual evidence that during ADT the iridium particles underwent surface oxidation with partial Ir dissolution. This is why the thin amorphous hydrous IrO(OH)_*x*_ layers on crystalline Ir particles are likely the location where iridium vacancies and redox-active electrophilic oxygen ligands—reactive intermediates and the very likely the catalytic active sites in IrO_*x*_ OER catalysts—can be suspected.^43^ It is known that the interaction between surrounding oxygen ligands and iridium centers adjacent to vacancies leads to significantly shortened, more covalent Ir–O bonds which favor the emergence of electrophilic O surface ligands (in the literature referred to as O^I–^) with an enhanced catalytic reactivity toward nucleophiles such as water. We will explore structural and electronic fingerprints of the catalytically active Ir–O metal–ligand bonds in the next section.

### 3.5 Exploring Chemical States, Bonds, and Performance Decays under Catalytic Operating Conditions

**Figure 7:**
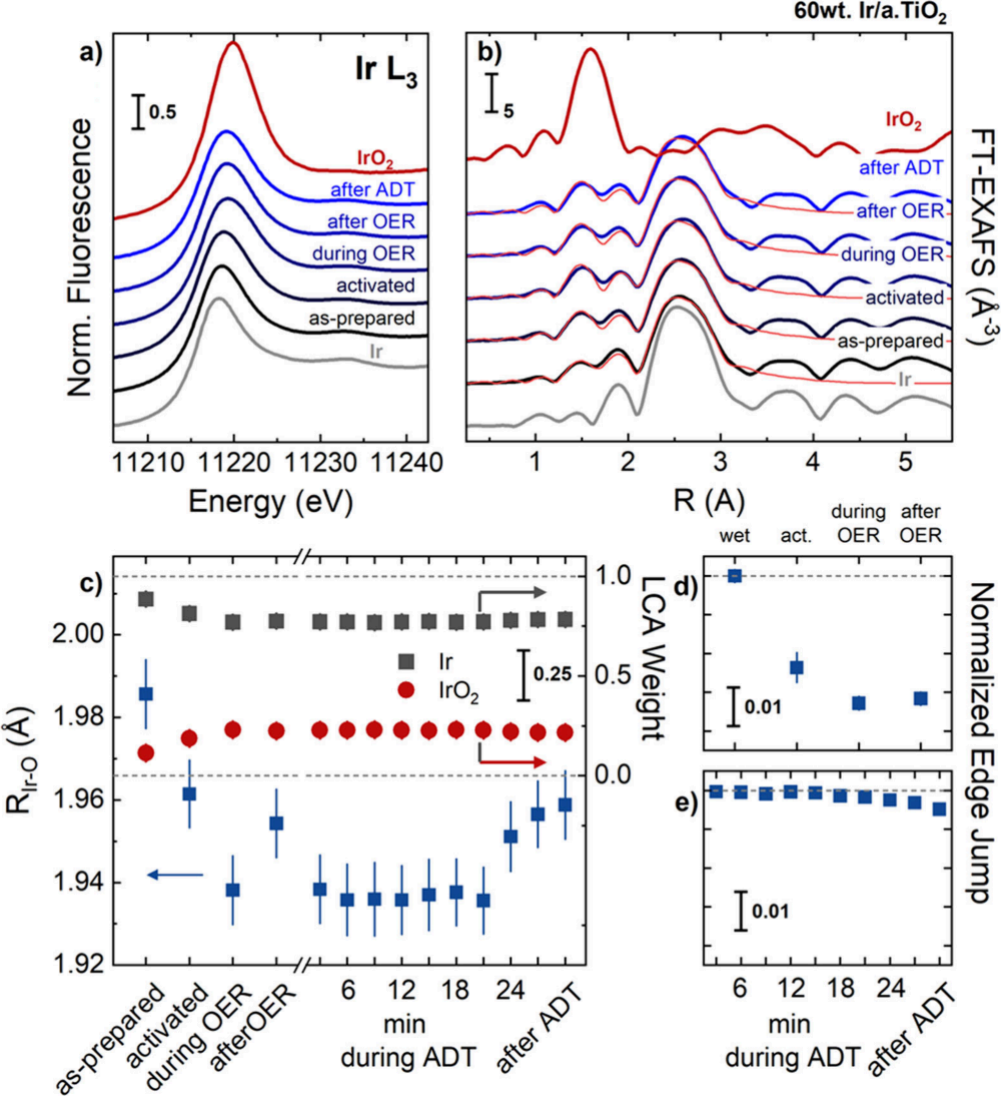
(a) Operando XANES and (b) Fourier-transformed EXAFS of 60% Ir/TiO_2_ recorded at the Ir L_III_-edge at different stages of electrocatalytic testing. The XANES profiles and FT-EXAFS of Ir metal (gray) and rutile IrO_2_ (red) reference materials are shown. The fits of the EXAFS data are shown in light red for the 60% Ir/a.TiO_2_ in panel (b). Panel (c) shows the linear combination analysis (LCA) of the XANES profiles with the Ir (gray) and IrO_2_ (dark red) reference data for the different electrochemical stages of the Ir 60% Ir/a.TiO_2_ as well as the fitted Ir–O distances as extracted from the EXAFS fitting. Panel (d) and (e) show the normalized edge jump of the Ir L_3_-edge during initial polarization as well as during the ADT with respect to the wet and the start of the ADT, respectively. The error bars in panel (c) refer to the standard deviation of fit and in panels (d)-(e) to the standard deviation of the average of several individual spectra. See Supplementary Figures S18 and S20 and Supplementary Table S3 for further details.

To study the formation of the active state and, in particular, the evolution of the local atomic structure of the Ir, we performed *operando* X-ray absorption spectroscopy. Figure 7a shows the Ir L_3_-edge XANES profiles of the 60% Ir/a.TiO_2_ catalysts in the as-prepared state, after activation (“activated” or AA), during and after OER, as well as after ADT. Ir and IrO_2_ reference spectra are provided. The as-prepared Ir L_3_-edge XANES is consistent with what is expected for a largely metallic Ir precursor material that gradually oxidized during the electrochemical activation. The oxidation is evidenced, in particular, by an increasing intensity of the white line (WL) at ∼11,218.5 eV and a slight shift to higher energies (Figure 7a). During the OER, the intensity of the Ir L_3_-edge further increased reversibly and the absorption edge shifted to higher energies, Supplementary Figure S19. This directly evidences the oxidation of the Ir NPs during the emergence of their catalytic active state and has been associated with the formation of covalent Ir^z+^–O^I–^ metal–ligand surface motifs under the catalytically active anode.^7,21^ Here, the O^I–^ represents a redox-active ligand with electrophilic character. The emergence of shared electron density between Ir and O thus tends to lower the Ir chemical state. We note that the accurate formal charge state of the Ir centers is irrelevant for the understanding of the phenomenon.

In addition to the XANES analysis, we performed extended X-ray absorption fine structure (EXAFS) analysis to obtain quantitative insights into the first coordination shells of the Ir atoms (Figure 7c, Supplementary Table S3). In agreement with the XANES profile, the Fourier-transform of the EXAFS (FT-EXAFS) are consistent with metallic Ir NPs with a coordination number (CN) of ∼6.8 ± 0.3 of the metallic Ir–Ir distances. After catalyst activation, the Ir–Ir CN decreases slightly to 6.3 ± 0.4, in line with the partial oxidation, the formation of IrO_2_-like species, detected by linear combination analysis (LCA) and with oxide shell-metal core structures of the Ir NPs found by STEM-EDX. At the same time, the Ir–O CN increased from 1.4 ± 0.2 to 1.7 ± 0.2. Furthermore, we identified a reversible contraction of the Ir–O during active state formation from 1.963 ± 0.008 Å after activation to 1.942 ± 0.008 Å during OER and back to 1.957 ± 0.008 Å after OER. This bond contraction reflects the reversible formation of more covalent Ir^z+^–O^I–^ species.^7,21^ We also determined the intensity of the as-measured Ir L_3_ fluorescence signal, and used it to track the loss of Ir on the electrode (Figure 7e). During the electrochemical activation, ∼2.5% of the Ir atoms are lost from the electrode, and the Ir signal decreased further by 0.8% during the OER. This loss of Ir atoms does not proceed while leaving the OER regime, as the edge jump did not decrease further after the OER. In contrast, we identified only a minor loss of Ir (less than 1%) during the first ADT, which is in agreement with the insights obtained from the online SFC ICP-MS investigations and confirms the enhanced stability of the electrocatalysts.

We then followed the chemical state and the local atomic bond length of Ir–O pairs under OER conditions during the ADT. Over the course of the ADT, the concentration profiles of oxidized and metallic Ir species, as determined from LCA, remained nearly constant (Figure 7c), even though the IrO_*x*_ NPs appeared slightly more metallic toward the end of the ADT. Similarly, the Ir–O distances, as determined from *operando* EXAFS data fitting, which initially matched those obtained during initial OER testing, grew gradually longer and ended up in a local structural (and presumably electronic chemical redox) state close to the “after OER” conditions. These findings confirm the underlying principle of the oxygen-evolving state of IrO_*x*_ NPs even on oxide supports and furthermore show remarkable stability even during the harsh pulsed treatment applied here.

### 3.6 Discussion of Reactivity and Stability of the 60% Ir/a.TiO_2_ Catalyst

To bring the individual experimental observations into a consistent picture of the relation between the structure, activity, and stability, we relate the initial XANES L_3_ edge decrease of 2.5% of the activated state of 60% Ir/a.TiO_2_ to the significant Ir dissolution spike in ICP-MS (Figure S8a). However, this dissolution and detachment of surface Ir were concomitant to a sharp enhancement in catalytic activity after activation (Figure S6a). The HR-STEM analysis (Figure 6a and 6b) provided visual evidence and insight into this coupled activity-stability event through the emergence of an Å-thick oxide layer with individual Ir atoms separated from the crystalline surface. This observation aligns with the prevalent catalytic activation mechanism of IrO_*x*_ surfaces,^20^ which suggests the formation of Ir vacancies and electrophilic O^I–^ oxygen ligands in their immediate proximity. Furthermore, this observation is further backed by the decrease in the Ir–Ir coordination number from 6.8 to 6.3 and the increase in the Ir–O coordination number from 1.4 to 1.7, (Figure 7). During the OER process, the Ir–O bond length decreased to 1.94 Å, which is shorter than the average Ir–O in crystalline IrO_2_, and similar to Ir–O distances in Ni-depleted IrNiO_*x*_ reported by Nong et al.^7^ The presence of covalent O^I–^ follows the number of Ir vacancies and is thought to account for the high OER activity observed in the 60% Ir/TiO_2_ catalyst in this study (Figures 3a, 3b and 4c). Despite its initial Ir dissolution, the 60% Ir/a.TiO_2__ADT catalyst exhibited only a fifth of the mass activity loss of the commercial reference catalyst. To rationalize this, additional microscopic (Figure 6g and 6h) and spectroscopic (Figure 7c) insights were needed. These analyses revealed that the oxygen-evolving process on IrO_*x*_ or hydrous IrO(OH)_*x*_ surface sites can occur simultaneously with the growth of an oxide layer.^44^ Within the oxide layers, the Ir–O distance undergoes a reversible contraction after OER, suggesting the reversible formation of catalytically active electrophilic Ir^z+^–O^I–^ pairs, the active centers in IrO_*x*_-based catalyst, after ADT. This reversible contraction ensures negligible losses of Ir NPs on the surface (lower than 1%) and retains the active state of Ir-surface sites during ADT. The remarkable stability of the Ir NPs on the surface of 60% Ir/a.TiO_2_ catalyst and the incompleted oxidation of the hydrous IrO(OH)_x_ during ADT may be attributed to the presence of catalyst–support interactions (Figure 4b), where the proximity of the oxide support impedes detrimental overoxidation of Ir.^45^

## 4 Conclusions

This work has explored the chemical bulk, surface characteristics, and surface electrocatalysis of a family of Ir/TiO_2_ OER catalysts. The Ir/TiO_2_ catalyst–support system is currently considered as the most promising anode catalyst materials to meet the physicochemical (e.g., electrical conductivity), catalytic OER reactivity, and chemical stability requirements of materials for low-Ir loaded PEM water electrolyzer anodes. The current work has identified a novel Ir/anatase TiO_2_ catalyst–support system that offers significantly enhanced OER performance. The catalyst demonstrates 5–7 times higher mass activity and 2–3 times higher specific activity in RDE measurements, and 36 times higher mass activity in MEA testing compared to the industry-standard material, with an optimal Ir loading of 60 wt %. The superior performance of the Ir/a.TiO_2_ catalyst, relative to a commercially available reference catalyst, was experimentally confirmed, even at substantially lower iridium loadings (at 0.3 mg_Ir_ cm^–2^). The catalyst features excellent conductivity and balanced stability and thus represents a promising low-Ir loaded anode catalyst candidate rivaling today’s commercial materials.

Detailed atomic scale imaging coupled to *operando* spectroscopic analysis of local bond structure of the 60% Ir/anatase TiO_2_ catalyst revealed unusual morphological and chemical stability of catalytically active hydrous IrO(OH)_*x*_ NPs on the TiO_2_ support. This stability is the manifestation of catalyst support interactions between the IrO_*x*_ particles and the TiO_2_ surface. Analysis of the catalytic active state of hydrous IrO(OH)_*x*_ NPs of the 60% Ir/anatase TiO_2_ catalyst revealed reversible Ir–O bond contraction resulting in electrophilic O^I–^ ligands, congruent with covalent Ir–O bonds reported in bimetallic bulk IrNiO_*x*_ catalysts. By contrast, the density of electrophilic O^I–^ ligands is correlated to the catalytic rate and thus, is likely substantially lower for the less defective bulk crystalline IrO_2_.

## Supporting Information Available

Methodologies, physicochemical characterization, electrochemical protocols, Supplementary Figures, and Supplementary Notes. (PDF)
