# Supplementary material for: Reactivity and Stability of Reduced Ir-Weight TiO2-Supported Oxygen Evolution Catalysts for Proton Exchange Membrane (PEM) Water Electrolyzer Anodes
Source: J Am Chem Soc. 2024 Nov 11;146(46):31444–55. doi: 10.1021/jacs.4c07002 (PMC11583366; doi:10.1021/jacs.4c07002)
Supplement: Supplementary file 1 — ja4c07002_si_001.pdf [file ja4c07002_si_001.pdf]

# *Supplementary Information*

## Reactivity and Stability of Reduced Ir-Weight $TiO_2$-Supported Oxygen Evolution Catalysts for Proton Exchange Membrane (PEM) Water Electrolyzer Anodes

*Hoang Phi Tran[a, b], Hong Nhan Nong[a], Matej Zlatar[c, d], Aram Yoon[e], Uta Hejral[e], Martina Rüscher[e], Janis Timoshenko[e], Sören Selve[f], Dirk Berger[f], Matthias Kroschel[a], Malte Klingenhof[a], Benjamin Paul[a], Sebastian Möhle[a], Kerolus Nasser Nagi Nasralla[a], Daniel Escalera-López[e], Arno Bergmann[e], Serhiy Cherevko[c], Beatriz Roldan Cuenya[e] and Peter Strasser[a]*.*

[a] Department of Chemistry, Chemical and Materials Engineering Division, The Electrochemical Energy, Catalysis and Materials Science Laboratory, Technische Universität Berlin, Straße des 17. Juni 124, 10623 Berlin, Germany

[b] Department of Chemical Engineering, Faculty of Physics and Chemical Engineering, Le Quy Don Technical University, 236 Hoang Quoc Viet, Bac Tu Liem District, Hanoi, Viet Nam.

[c] Forschungszentrum Jülich GmbH, Helmholtz-Institute Erlangen-Nürnberg for Renewable Energy (IET-2), Cauerstraße 1, 91058 Erlangen, Germany

[d] Department of Chemical and Biological Engineering, Friedrich-Alexander-Universität Erlangen-Nürnberg, Egerlandstr. 3, 91058 Erlangen, Germany

[e] Department of Interface Science, Fritz-Haber-Institute of the Max-Planck-Society, Faradayweg 4-6, 14195 Berlin, Germany

[f] Center for Electron Microscopy (ZELMI), Technische Universität Berlin, D-10623 Berlin, Germany

***pstrasser@tu-berlin.de**

## Supplementary Methods

### Synthetic protocols

In the following we describe the synthetic procedure for the preparation of various Ir loadings (x wt %) on anatase $TiO_2$ (x ranging from 30% to 80%). Approximately 1.22 mg of dihydrogen hexachloroiridic acid ($H_2IrCl_6 \cdot 6H_2O$, with an Ir content of 35-42% (sourced from Sigma Aldrich) was dissolved through agitation in 1.08 L of deionized water ($H_2O$). The precise concentration of $Ir^{+4}$ was subsequently determined using ICP-OES. Afterward, approximately 100 mL of the resulting precursor solution was added to a specific quantity of anatase $TiO_2$ (a.$TiO_2$), which had been pre-dispersed in isopropanol (IPA) using a horn-sonifier for a duration of 30 minutes. The quantity of a.$TiO_2$ used was calculated to achieve the desired Ir loading, ranging from 30 wt % to 80 wt%, in the final product. The samples were denoted as x% Ir/a.$TiO_2$, where x represents the weight percentage of iridium on anatase titania. The catalysts were finalized by a thermal treatment under synthetic air flow, at 300 °C, in 1 hour.

### Physico-chemical Characterization

***Crystalline structure.***

X-ray diffraction (XRD) patterns were recorded utilizing a D8 Advance X-ray diffractometer (Bruker AXS) that was equipped with a Cu Kα radiation source, a variable divergence slit, and a position-sensitive detector. Data acquisition was conducted over a 2θ range spanning from 10° to 95°, with a measurement step size of 0.06°. Each measurement required 6 seconds per step, and there was no sample rotation during the data collection process.

The data analysis was performed using Origin 2021b software (© OriginLab Corporation). To determine the size of the crystalline particles, the full width at half-maximum (FWHM) of the Bragg reflections was measured and adjusted to account for instrumental line broadening effects.

The Scherrer equation was subsequently applied in the analysis, utilizing a Scherrer constant value of 0.89.

***Elemental analysis via inductively coupled plasma – optical emission spectroscopy (ICP-OES)***

*Sample preparation*

A precise procedure was followed: Initially, 40 mg of sodium chlorate ($NaClO_3$) was added to approximately 10 mg of the unannealed catalyst, which comprised both unsupported and supported catalyst samples. This mixture was then carefully transferred into a vessel made of Polytetrafluoroethylene (PTFE-TFM). Subsequently, 8 mL of concentrated Aqua Regia was added drop by drop while being stirred meticulously, ensuring that the catalyst powder was exposed to the acidic solution uniformly.

The resulting mixture was allowed to rest in the PTFE vessel at room temperature for 2 hours to allow for the release of gases. To prevent contamination by airborne dust, the vessel was covered with a paraffin film during this period.

A Microwave Digestion System, provided by Anton Paar, was employed to conduct the chemical reaction. The reaction was carried out at a temperature of 200°C for a duration of 20 minutes, with a heating ramp of 10 minutes.

Following the reaction, the obtained sample was allowed to cool down to room temperature, after which it was filtered to remove any solid particles. It was then diluted and adjusted to a final volume of 50 mL using ultrapure water. A 1 mL aliquot of this solution was further diluted by a factor of 10 before being transferred into a plastic ICP-tube, where it was stored for subsequent analysis via Inductively Coupled Plasma Optical Emission Spectroscopy (ICP-OES).

*ICP-OES measurement*

Elemental analysis was conducted using an ICP – OES analysis system, specifically the 715-ES model by Varian. To ascertain the concentration of iridium, a series of standard solutions with Ir

concentrations of 0.5, 1.5, 3, 4.5, and 6.0 ppm were prepared. The precise Ir concentration was determined by the intensity of emitted light at multiple selected wavelengths, including 212.681, 224.268, 236.804, 254.397, and 263.971 nm.

***Elemental Analysis using X-ray fluorescence (XRF):***

We employed a high-performance wavelength dispersive XRF spectrometer, the S8 TIGER by Bruker AXS, for the semi-quantitative determination of the elemental composition within the support materials, unsupported catalyst, and supported catalyst powders. Each sample, taking approximately 20 mg, was meticulously prepared by placing it in 6 mm XRF open-ended aperture cups provided by Chemplex Industries. These cups' bottoms were then sealed with a Prolene® film, measuring 2.8 μm in thickness. Subsequently, these prepared samples were inserted into a 5 mm automatic mask sample holder, designed by Beamguide, and loaded into the sample loaders.

It's crucial to emphasize that this method provides accurate quantification of metallic elements present in the supported catalyst components but does not yield information about the oxygen content or the oxidation state of the metal within the samples. Consequently, when comparing samples using this technique, the atomic ratio of metallic elements, such as Ir:Ti, offers a more precise measure than the absolute calculated weight percentage of each element. To ensure reliability, each measurement was conducted three times to ascertain an error margin.

The composition of the powder samples was analyzed using an S8 Tiger XRF-spectrometer, also from Bruker AXS in Germany, and the QUANT-EXPRESS calibration method was applied.

***Elemental Analysis using transmission electron microscopy (TEM) and energy dispersive X-ray spectroscopy (EDX).***

TEM micrographs were acquired using a FEI TECNAI G2 20 S-TWIN microscope equipped with a LaB6 cathode and a GATAN MS794 P CCD camera. The microscope operated at an acceleration voltage of 200 kV. To ensure the absence of organic compounds, as-prepared catalyst powders were suspended in water using horn-sonication in a 6 mL glass vial, resulting in a light

grayish suspension. Subsequently, 10 μL of this suspension was pipetted onto a carbon-coated copper grid (400 mesh, Plano) and dried for 15 minutes at 70°C.

TEM is frequently coupled with EDX to ascertain the bulk composition of a sample. Various scanning procedures, including box, line, and elemental maps, offer more detailed insights into homogeneity and particle type. EDX mapping analysis conducted during the initial characterization, after activation, and post-stability tests can yield vital information concerning dissolution, particle distribution, and segregation.

EDX analysis was performed using an r-TEM SUTW Detector (EDAX Inc., NJ, USA) equipped with a Si(Li) detector, providing an energy resolution lower than 136 eV for Mn Ka, with detection capabilities extending from Boron (Z=5). Alloy and support size distributions were determined by measuring around 300 particles, and the data were analyzed using ImageJ 1.48 software developed by the U.S. National Institutes of Health.

***Zeta-potential (ζ)***

*Sample Preparation:*

About 10 mg of the catalyst powder was dispersed in ultrapure water (Millipore, 18 MΩ) to create a 100 ppm suspension. This suspension underwent 30 minutes of horn-sonication, with the vial cooled using ice during sonication to prevent solvent evaporation and ensure the formation of an ink-like suspension.

*Zeta-Potential Measurement:*

The surface charge behavior of metal and metal oxide nanoparticles (NPs) under different conditions was determined by the titration curves, using a Malvern Zetasizer Nano ZS.

For the measurements, a 100 ppm ink was prepared in three different media: (1) water dispersion, (2) water dispersion adjusted to pH 12.5 using 0.1 M NaOH, and (3) dispersion in 1 M $NaNO_3$ adjusted to pH 12.5 using 0.1 M NaOH. The pH titration curve of the zeta potential was conducted to determine the initial pH of the dispersed particles and the pH of the isoelectric point (IEP). This

titration-measurement process was repeated until a pH of 1.5 was reached, utilizing a Disposable Folded Capillary (DFC) cell.

The Smoluchowski model was selected for F(κa) calculation, and the Monomodal model was employed for analysis.

The zeta potential titration curves of a.$TiO_2$ are illustrated in **Figure S16**.

***Conductivity measurement.***

The electrical conductivity of both the support material and the supported iridium-based catalyst powder were assessed using the apparatus illustrated in **Figure S2**. The design of this conductivity tester drew inspiration from various designs previously documented in the literature.[5, 6] It comprised a 10 mm diameter cylinder made of Soda glass, with a centrally bored hole of 0.8 mm diameter. This glass cylinder was positioned between two brass pistons. A specific quantity of powder was weighed and placed inside the glass cylinder between the two brass pistons. The applied pressure (in range of 40 – 200 MPa), measured by a precision balance integrated into a 2-pillar manual press system, was applied to the top piston using a twist pressing wheel. Additionally, the weight of the upper piston was calibrated prior to measuring the applied pressure on the powder:

$$\sigma = \frac{h}{R} \times \frac{1}{A} \qquad (1)$$

Where σ is the electrical conductivity (S $cm^{-1}$), $R_h$ is the electrical resistance of the compressed powder, $R_0$ is the electrical resistance of the system without powder (thus $R = R_h - R_0$) (Ω), h is the thickness (cm), and A is the cross sectional area ($cm^2$) of the powder under compression.

## Electrochemical measurements

### *Electrode preparation*

The deposition of the Ir x wt % on a.$TiO_2$ catalyst onto a meticulously polished and cleaned 5 mm-diameter Au working electrode (WE) of a rotating disk electrode (RDE) (Pine Research Instrumentation) was carried out using the drop-coating method. To create the catalyst ink, a specific quantity (5 - 10 mg) of the supported Ir-based catalyst powder was combined with a Nafion solution (5 wt % in lower aliphatic alcohols and $H_2O$, Sigma-Aldrich), which contained 25% of Nafion by weight relative to Ir.

This mixture was dispersed using horn-sonification for 30 minutes in a solution of 2-propanol and Milli-Q water, maintaining a precise volume ratio of 1:4 to attain a precise concentration of 0.392 mg $ml^{-1}$ of Ir. Subsequently, a carefully controlled 10 μL of the resulting ink was drop-coated onto the prepared gold electrode surface and allowed to dry for 8 minutes at 60°C under ambient air conditions. The amount of Ir loaded onto the gold electrode surface was meticulously adjusted to precisely 3.92 μg, which corresponds to an approximate loading of 20 μg $cm^{-2}$.

### *Electrochemical measurements*

For all electrochemical measurements, a RDE configuration was employed. This setup featured a three-compartment electrochemical glass cell, complete with a Luggin capillary, and operated under RT conditions. The electrolyte used throughout these experiments was a 0.05M $H_2SO_4$ solution, which was saturated with nitrogen gas.

In these experiments, a 5 mm-diameter gold electrode served as the WE, while the reference electrode (RE) utilized was Mercury/Mercurous Sulfate (MMS). The MMS reference electrode was meticulously calibrated against a reversible hydrogen electrode (RHE) within the same electrolyte. A clean platinum mesh counter electrode (CE) was employed. It is noteworthy that all potential values mentioned in this article are reported with respect to the RHE.

*Electrochemical oxidation*[1, 2]

The working electrode potential was cycled between +0.05 V and +1.50 V for 50 cycles at a scan rate of 500 mV $s^{-1}$ in 0.05M $H_2SO_4$ aqueous solution to oxidize the metallic Ir to form $IrO_x$ on the oxide support surface, the activated catalyst for the oxygen evolution reaction (OER), and stabilize the OER catalytic activity.

*Electrochemically accessible Ir oxide active surface evaluation*[1, 3]

To estimate the accessible Ir oxide active surface, the working electrode potential was scanned between +0.4 V and +1.3 V at a scan rate of 20 mV $s^{-1}$ for 3 cycles. The electrochemical active surface area (ECSA) of the supported Iridium-based catalyst was calculated by integrating the anodic charge from the anodic sweep of the 3$^{rd}$ CV cycle.

*Electrocatalytic OER protocol. (OER test protocol)*[1, 3]

Following the determination of the apparent electrochemical surface area (ECSA), the electrochemical catalytic activity of Ir NPs on a.$TiO_2$ was assessed and subsequently normalized for comparative analysis. To account for Ohmic losses (iR), Potential Electrochemical Impedance Spectroscopy (PEIS) measurements were conducted for correction.

Subsequently, polarization curves were generated by scanning the potential from 1.0 V to 1.8 V in the anodic direction and from 1.8 V to 1.0 V in the cathodic direction. These scans were performed at a scan rate of 5 mV $s^{-1}$ and a rotation speed of 1600 rpm. Based on these polarization curves, three distinct current densities were determined: Geometric current densities ($j_{geo.}$), normalized to the geometric area of the gold (Au) electrode (~0.196 $cm^2$). Ir-mass based OER current densities ($j_{mass}$), normalized to the actual amount of Ir loaded on the electrode surface. Specific current densities ($j_{spec.}$), normalized to the anodic charge ($q_A$*) measured between 0.4 V and 1.3 V at a scan rate of 50 mV $s^{-1}$.

The OER current was calculated by averaging the current values obtained from the iR-corrected anodic and cathodic scans at an overpotential of 300 mV on the polarization curves.

*Accelerated degradation test.*

All Rotating Disk Electrode (RDE) measurements were carried out in a 0.05 M $H_2SO_4$ solution at room temperature. The measurements were conducted using a SP200 Potentiostat (BioLogic, France).

Electrodes were immersed in a degassed electrolyte (15 min, 1600 rpm, under $N_2$ flow), followed by initial characterization with CVs scans and OER performance test as described above. In accordance with a transient accelerated degradation test (ADT) outlined in the literature,[4] the following steps were undertaken: : In total 5,000 cycles of square-wave voltammetry (SQWV) was measured between a lower limit potential, $E_{LLP}$ = 1.23 $V_{RHE}$, and an upper limit potential $E_{ULP}$ = 1.60 $V_{RHE}$. Each potential was held for three seconds per cycle. Every 1,000 cycles the SQWV was interrupted for electrochemical impedance spectroscopy (EIS), three CVs between 0.4 – 1.4 $V_{RHE}$ (50 mV $s^{-1}$), and a slow OER scan (5 mV $s^{-1}$, 1.00 $V_{RHE}$ to 10 mA $cm^{-2}$). After the ADT section, the initial OER scans and CVs were repeated.

EIS was measured at 1.0 $V_{RHE}$ with a single sine amplitude of 10 mV from 200 kHz to 1 Hz with 10 points per decade and 3 repetitions per scan.

**PEM-WE Measurement**:

The membrane electrode assembly (MEA) for PEM single cell measurements was prepared in a catalyst-coated membrane (CCM) configuration, using a Nafion NR-212 membrane with a total active area of 5 $cm^2$. CCMs were tested using a Greenlight ETS electrolysis test station (Greenlight, Canada) at 80 °C and 1 bar balanced pressure.

For the anode, a specified amount of catalyst powder was suspended in a water/isopropanol mixture with 13 wt% solid ionomer content (Nafion solution, 5% wt, Sigma Aldrich) based on

total solid content (solid ionomer and catalyst). This translates into an ionomer to catalyst (I/C) weight ratio of 0.15 for both the 60 wt% Ir/a.$TiO_2$ catalyst (60 $wt\%_{Ir}$) and the Umicore Elyst 75 reference catalyst (75 $wt\%_{Ir}$ ). This suspension was sonicated in an ice bath for 30 minutes. The resulting ink was then sprayed onto a fluorinated ethylene propylene (FEP) decal foil using an automated ultrasonic spray-coater (Sonotek).

Cathodes were prepared by roll-milling a measured amount of catalyst (Pt/C, Umicore Elyst 50) with isopropanol, water, and ionomer (Nafion, 20 wt%, Sigma Aldrich) overnight, maintaining an ionomer to carbon weight ratio of 0.12. The resulting slurry was bar-coated onto a Kapton foil decal using a film applicator (BEVS Co., Ltd.) and then cut using an automated cutting plotter. Both the anode and cathode layers on their respective decals were then transferred onto the ionomer membrane via a hot-pressing decal-transfer process (155 °C, 2.4 MPa, 3 min). Catalyst loadings (0.3 $mg_{Ir}cm^{-2}$ 60 % Ir/a.$TiO_2$, 1.39 $mg_{Ir}cm^{-2}$ and 0.29 $mg_{Ir}cm^{-2}$ Umicore Elyst 75) were determined by weighing the decal before and after the transfer process.

The cell assembly involved a titanium-based porous transport layer (Bekaert, 150 μm) sputtered with platinum on the anode side, and a carbon paper with a microporous layer (Sigracet 22 BB, 190 μm) on the cathode side. The cell was operated at 80 °C with an anode water feed of 50 mL $min^{-1}$ at atmospheric pressure. A current hold of 1 A $cm^{-2}$ was employed as a short break-in protocol. Polarization curves were recorded under galvanostatic control by incrementally adjusting the current. The voltage at each step was calculated by averaging the values from each current step. The high-frequency resistance (HFR) was determined at each step using galvanostatic impedance spectroscopy. Ir demands were determined at about 1.78 V cell voltage ( 70% efficiency LHV) using the uncorrected Polarization data and the geometric Ir loadings.

### ***Advanced characterization methods***

*High Resolution Scanning transmission electron microscopy (HR-STEM)*

We conducted scanning transmission electron microscopy (STEM) and energy dispersive X-ray spectroscopy (EDS) using specialized equipment. STEM imaging was carried out with a FEI TITAN 80-300 electron microscope, which operates at 300 kV and is equipped with an Image Cs corrector (CEOS) and a high-angle annular dark-field (HAADF) detector. HR–STEM and elemental mapping were performed using a Jeol JEM-ARM300F2, a probe aberration-corrected 300 kV STEM/TEM with a cold field emission electron source and JEOL ETA Probe Cs corrector. The JEM-ARM300F2 offers a STEM DF resolution of 63 pm at 300 kV and 110 pm at 80 kV for HAADF imaging.

EDX maps were recorded using a windowless dual SDD detector system (Jeol) with dimensions of 2 x 160 $mm^2$ and a solid angle of 2.2 sr along with the instrument provided software AnalysisStation (Jeol). The sample was dispersed in milliQ-water and drop-deposited onto lacey carbon-coated copper support grids.

*Scanning flow cell coupling inductively coupled plasma mass spectroscopy (SFC – ICP MS):*

Dissolved metal ions were quantified through the coupling of ICP-MS with a Scanning Flow Cell. This analysis was conducted in a 0.05 M $H_2SO_4$ solution purged with argon, achieving a detection limit for Ir in the range of 0.1 to 1 ppt. The electrochemical setup featured a graphite rod as the counter electrode and an Ag/AgCl electrode (Metrohm) as the reference electrode.

To prepare the electrolyte, highly concentrated sulfuric acid ($H_2SO_4$ 98%, Merck) was appropriately diluted with ultrapure water (PureLab Plus system, Elga, 18 MΩ cm, Total Organic Carbon (TOC) < 3 ppb). The flow rate through the cell was maintained at 352 μL $min^{-1}$. The stable performance of the ICP-MS instrument (NexION 300X, Perkin Elmer) was guaranteed through daily calibration and the addition of an internal standard solution downstream from the flow cell,

with measurements including isotopes: $^{187}Re$, $^{45}Sc$. A detailed protocol can be found in **Figure S17**.

For the electrochemical measurements, a potentiostat (Reference 600, Gamry) was employed.

The Stability numbers (S-numbers) were calculated based on the ratio between the amount of produced oxygen – calculated from total charge, generated during the ADTs, which was determined by integrating only the anodic charge at 1.55 $V_{RHE}$ pulses, versus dissolved iridium occurring during each ADT.

## Supplementary Figure

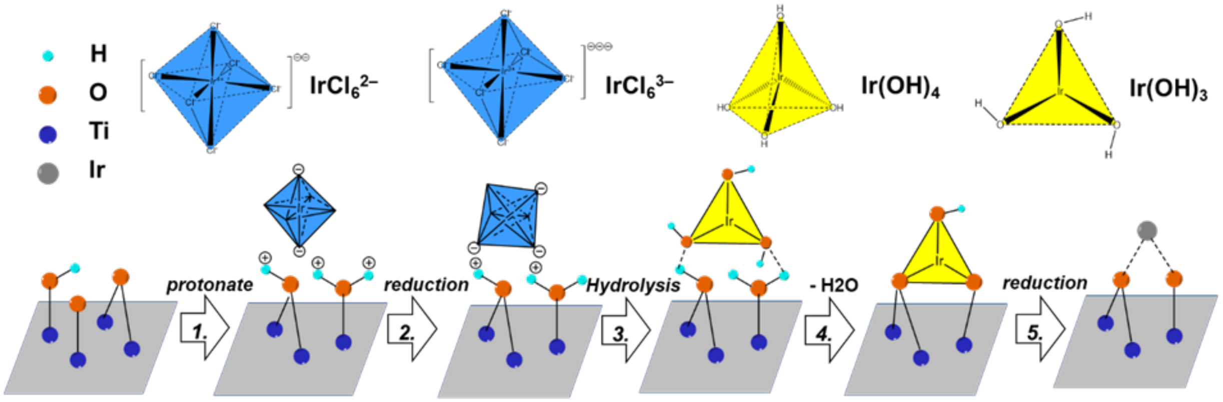


**Figure S1.** Suggested formation mechanism of iridium NPs deposited on anatase titanium dioxide from dihydrogen hexachloroiridate(IV) hydrate, or hexachloroiridic acid precursor: ***Step 1.*** Protonation of titania surface. ***Step 2.*** Anion $IrCl_6^{2-}$ adsorbed onto protonated $TiO_2$ surface was reduced to form $IrCl_6^{3-}$. ***Step 3.*** Hydrolysis of $IrCl_6^{3-}$ in alkaline media to form $Ir(OH)_4$, deposited on oxide support. ***Step 4.*** dehydration of $Ir(OH)_3$ with $H^+$ on the surface to form Ir (hydro)oxide sharing oxygen with Titania surface. ***Step 5.*** Deformation and reduction of iridium from Ir (3+) to metallic Ir (0) by isopropanol alcohol (IPA).

### *Supplemental Note 1:* Surface Interaction Synthesis of Ir-based catalyst on $TiO_2$ support

In the current synthetic process, the electrostatic interaction between the hexachloroiridate anion ($IrCl_6^{2-}$) precursor and the oxide support surface ($TiO_2$) plays a pivotal role in improving catalyst distribution. Therefore, comprehending the surface charge behavior and the Point of Zero Charge (PZC) of the support, as depicted in **Figure S16** and Supplemental Note 4, can yield valuable insights for optimizing synthetic conditions and selecting suitable precursors.

As illustrated in **Figure S1**, we have chosen hexachloroiridic acid, $H_2IrCl_6$, as the precursor of choice. This compound not only provides an acidic medium but also supplies iridium in the form of the anion hexachloroiridate, $IrCl_6^{2-}$. In a strongly acidic environment, the surface of a.$TiO_2$ carries a positive charge, serving as nucleation sites that attract the negatively charged $IrCl_6^{2-}$ ions. These ions can be partially reduced by IPA, forming the initial layer of iridium NPs.

Following anion adsorption, the pH of the reaction medium gradually increases during step 3, leading to the deposition and precipitation of $IrCl_x(OH)_{6-x}$. Subsequently, in step 4 and step 5, these

compounds slowly decompose and reduce into $Ir(OH)_3$ and then $IrO_x$ ($x < 2$) or metallic iridium when treated with IPA at 80°C. The deposition and precipitation processes occur on the surface of a.$TiO_2$, maintained at a pH of 8 to minimize repulsion forces between the as-prepared nanoparticles, thereby maximizing the deposition yield of the iridium NPs.

Throughout the experiments, the volume ratio of water, isopropanol, and 0.1M KOH was consistently maintained at 4:2:1, while the amount of titanium dioxide was varied to achieve specific loading content of Ir on a.$TiO_2$, ranging from 30 wt % to 80 wt%. The combined mechanisms of anion adsorption and deposition precipitation facilitate a homogeneous distribution of the initial layer at lower loading and enhance the deposition yield at higher loading.

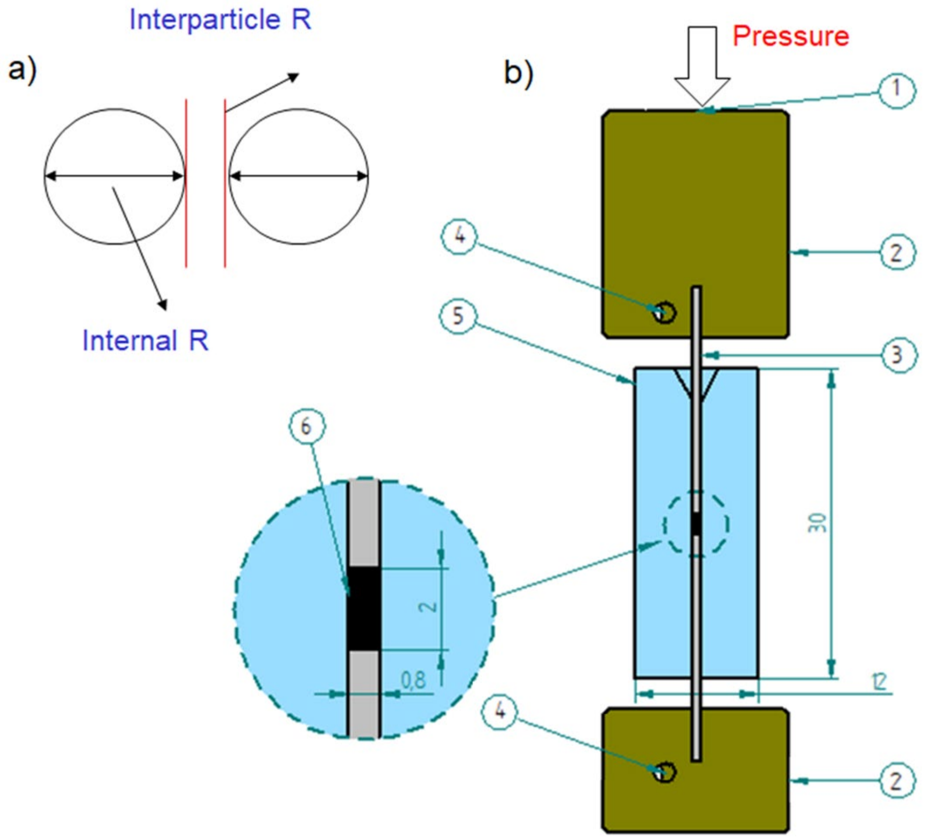


**Figure S2.** Experimental apparatus for measuring resistivity of compressed powders: a) Simplified model to estimate powder resistivity, as a combination of internal and interparticle resistivity and b) The experimental device comprising: 1. compression pressure, applied by a twist pressing wheel, 2. brass pistons, 3. hard steel tips, 4. holes for the banana plugs to connect to the potentiostat, 5. A 12 mm diameter sodaglass cylinder with a centrally bored hole of 0.8 mm diameter, 6. compressed powder (h from 3 to 9 mm, depending on powder density).

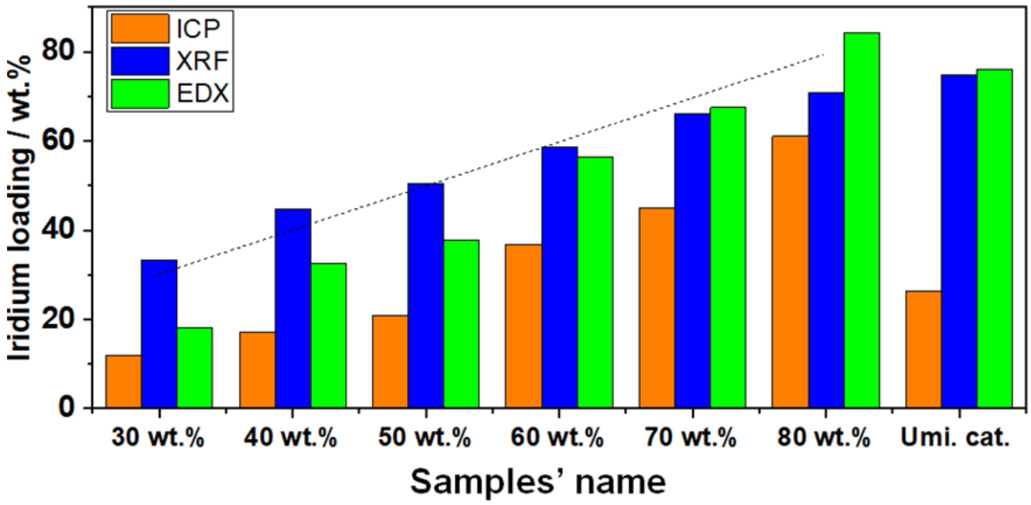


**Figure S3.** Comparison of iridium contents determined by three most popular elemental analysis methods, including ICP-OES (orange bars), XRF (blue bars) and EDX (green bars), for various synthesized Ir/a.$TiO_2$, and the commercial Umicore catalyst. The nominal Ir loading content expressed via the dot line.

**Supplemental Note 2: Physico-chemical Characterization**

When comparing the results from different elemental analysis methods, it was observed that XRF analysis provided values that were the closest to the nominal contents, especially in the 30-60 wt % range. Above 60 wt%, ICP-OES results showed significant improvement but still remained lower than those obtained from XRF analysis. Notably, there was a clear indication of incomplete dissolution of the supported catalysts in Aqua Regia during ICP-OES sample preparation, particularly at low Ir contents. This phenomenon, especially after thermal treatment, may lead to the underestimation of Ir contents using ICP-OES. Consequently, the observed differences can be attributed to the role of $TiO_2$ in enhancing the corrosion tolerance of Ir-based catalysts in acidic media.

In contrast, EDX provided relatively good results but only offered localized information. On the other hand, XRF analysis identifies elemental composition using characteristic X-rays, making it

a non-destructive, precise, and rapid analytical method. Furthermore, XRF elemental analysis provides the most reliable composition information, independent of solubility and locality considerations. As a result, the catalyst compositions determined via XRF will be used for Ir-normalization in all catalytic ink preparations in this study before electrochemical characterization.

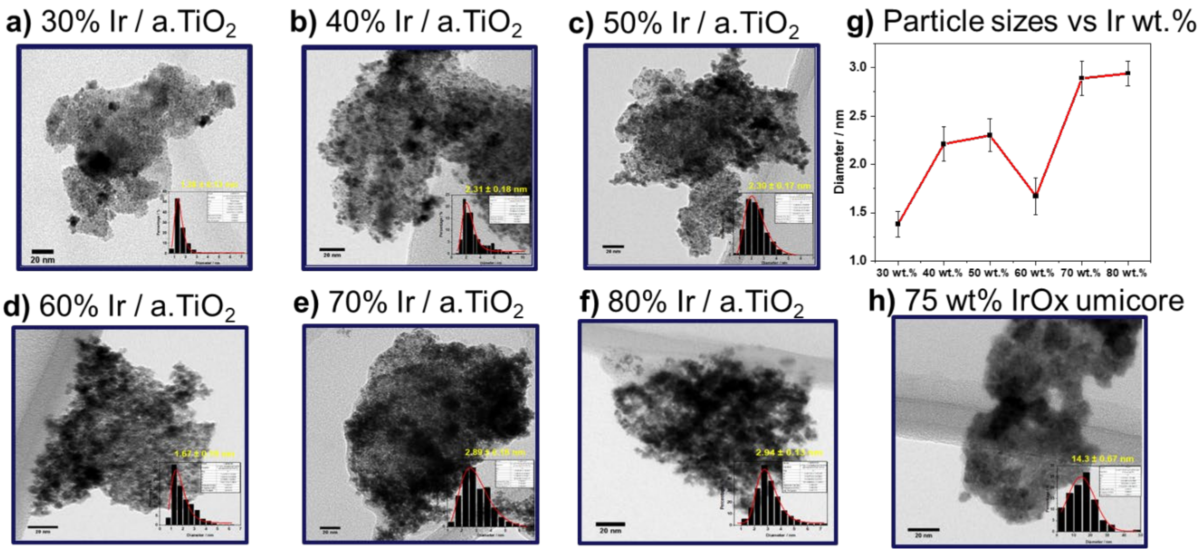


**Figure S4.** TEM images and particle sizes of x% Ir/a.$TiO_2$ at different Ir weight loadings. a) to f) TEM images displaying various Ir-loading contents, ranging from 30 wt % to 80 wt%, on a.$TiO_2$. The inset of each TEM image shows the particle size distribution histogram used for particle size calculations. g) Correlation plot illustrating the relationship between the calculated average particle sizes and the nominal Ir weight loading. h) TEM image showing the reference catalyst with a 75 wt % of Iridium (in oxide form) on $TiO_2$.

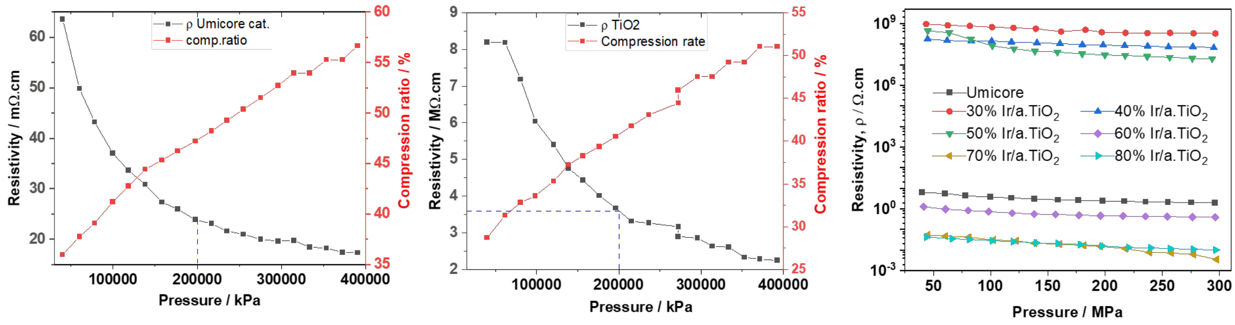


**Figure S5.** Analysis of resistivity and compression rates of different nano-powders in relation to compressing pressure: a) Correlation curves illustrating the relationship between resistivity, compression ratio, and compression rate for the Umicore Elyst catalyst. b) Profiles depicting the resistivity and compression ratio relative to the compression rate for anatase $TiO_2$ (sourced from Alfa Aesar). c) Pressure-dependent curves were constructed to investigate the resistivity of various Ir-content-supported Ir catalysts in comparison to the Elyst Umicore catalyst.

## Supplemental Note 3: Resistivity measurements

The measured resistivity of the powder gradually decreases asymptotically toward its intrinsic value during compression. However, the compression of the powder falls short of achieving the packing density of its crystalline structure. Nonetheless, under operation conditions, air voids and gaps between the closest grains and particles are filled with a conductive electrolyte. Consequently, interparticle resistivity, measured at high compression rates, offers more meaningful insights for further investigation of catalytic performance.

However, depending on the material's elasticity, hardness, compressibility, compactability, and particle arrangement, extreme compression can lead to particle rearrangement, damage, and distortion through elastic or plastic transformation. To facilitate comparison, resistivity

measurements at 200 MPa (~10 kgf 0.005 $m^{-2}$) were employed for various synthesized catalysts and the reference powders, including $TiO_2$ from Alfa Aesar and the Umicore catalyst.

As shown in **Figure S5a**, increasing the compression pressure from 0 to 400 MPa results in a 37% increase in the compression ratio of metallic iridium, while the resistivity decreases by a factor of 7, reaching approximately 0.20 Ω cm. In contrast, due to its differing softness, the compression ratio of a.$TiO_2$ only increases by 23%, and the resistivity decreases by a factor of 4, reaching around 2.106 Ω cm. To prevent heating or electron excitation of a.$TiO_2$, the applied potential must be kept at a low range. Consequently, the recorded currents are significantly low, requiring the use of the Keithley Sub-Femtoamp, **Figure S15c**, a more sensitive ammeter, for measurements.

Must notice that, for the high conductivity (low resistivity) materials, the measurable resistivity range is from $10^{-2}$ to 10 Ω cm, which can be recorded by the Gamry Preference 3000 Potentiostat/Galvanostat/ZRA, **Figure S15a**. This range is suitable for fitting with a simple circuit, with an error margin of less than 1 μΩ cm, which is lower than 10% of the measured value.

In contrast, for low conductivity (high resistivity) materials, the measurable resistivity range is from 0.1 to $10^2$ MΩ cm. Within this range, at an applied potential of just 1–1.5 V, the measured current is extremely low, which seems to be impossible to record using a standard Gamry potentiostatic/galvanostatic instrument. Specifically, in this range, the intrinsic resistivity is much higher than the interparticle resistivity, and the acceptable error can be in kΩ cm (around 10%). Thus, using a multimeter capable of detecting sub-femtoamp currents is more appropriate. And the recorded value must be an average, measured once every 5 seconds for 2 minutes.

The resistivity of the $TiO_2$ support is approximately 10 million times higher than that of $IrO_2/TiO_2$ Umicore. Consequently, when plotting all correlation curves together, as shown in **Figure S5c**, the observed variation in a single curve is negligible.

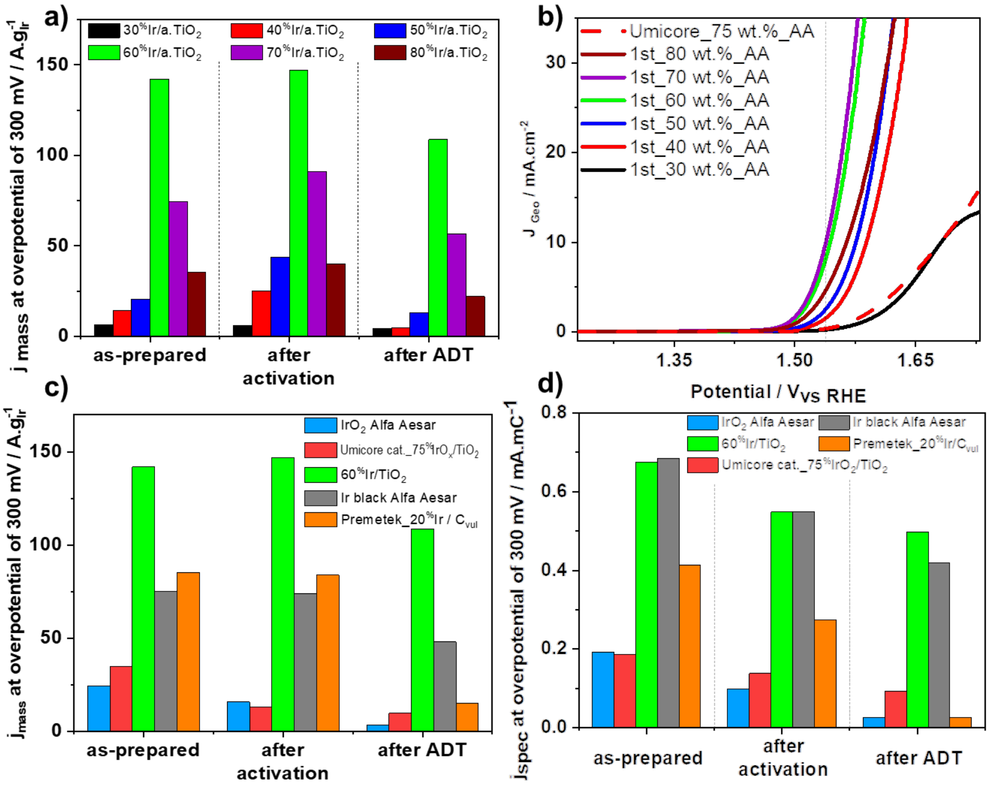


.

**Figure S6.** Activities and stabilities of various supported and unsupported Ir-based catalysts, are presented in the performance and stability of various Ir-based catalysts after activation and 5000 duty cycles of accelerated durability testing (ADT) from 1.23V to 1.6V states. a) Mass activity ($j_{mass}$) and b) polarization curves of geometric activity ($j_{geo}$) for different Ir loading contents on an a.$TiO_2$ support. Additionally, the performance of the best-synthesized catalyst, 60% Ir/a.$TiO_2$, is included for comparison. c) Mass activity ($j_{mass}$) and d) specific activity ($j_{spec}$) for various reference Ir-based catalysts, including Ir black (gray), $IrO_2$ (cyan) from Alfa Aesar, the Elyst Catalyst (fuchsia) from Umicore, and 20% Ir on carbon Vulcan (orange) from Premetek. Oxygen evolution reaction (OER) current densities were evaluated at an overpotential of 300 mV and normalized either for the applied Ir amount ($j_{mass}$) or for the anodic charge q* ($j_{spec}$). The rotating disk electrode (RDE) measurements were conducted under the following conditions: gold (Au) electrode, 0.05 M $H_2SO_4$, and a rotation speed of 1600 rpm. The Ir loading was approximately 20 µg $cm^{-2}$.

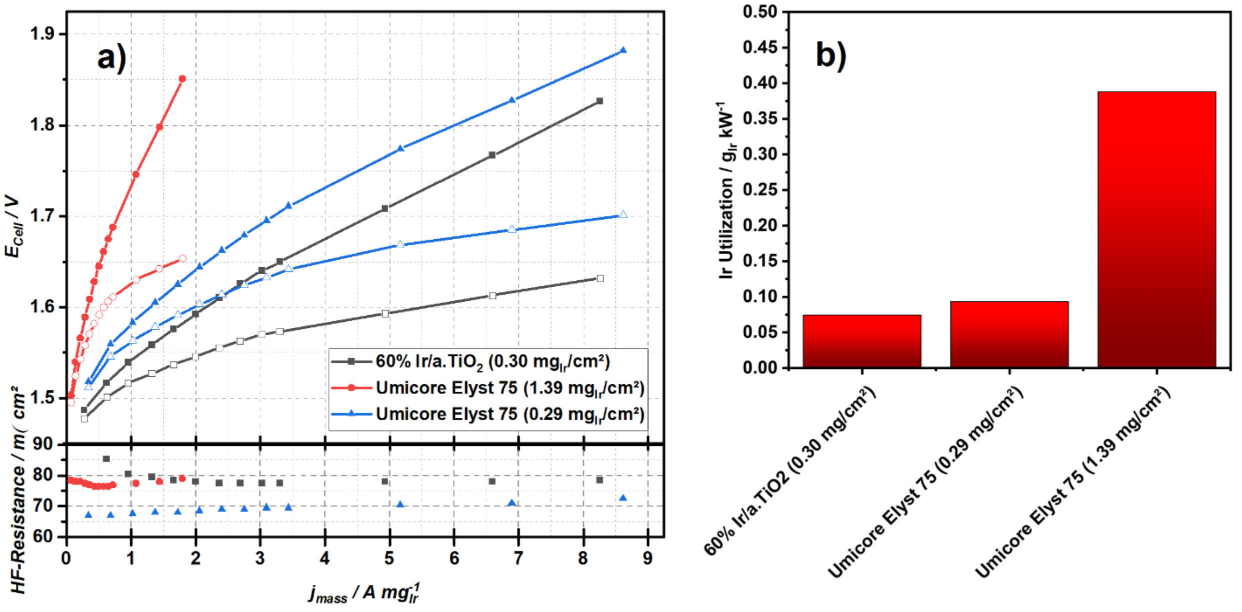


**Figure S7.** a) Mass normalized polarization curve of 60% Ir/a.$TiO_2$ together with a commercial iridium oxide reference at different loadings (Umicore Elyst 75; 1.39 $mg_{Ir}$ $cm^{-2}$; 0.29 $mg_{Ir}$ $cm^{-2}$). The HFR-corrected values are plotted using hollow symbols. Lines to guide the eye were added in order to increase the clarity of the plot. Measurement parameter: Both cathode (Umicore Elyst 50, 0.11 $mg_{Pt}$ $cm^{-2}$) and the iridium catalyst were coated onto the membrane (Nafion NR 212, 5 cm²) using the decal transfer process. Carbon paper (Sigracet 22 BB) was used as the gas diffusion layer on the cathode side, while Pt-sputtered titanium felt was used as porous transport layer on the anode side. The used torque was 35 Nm. An anode flow of 50 mL $min^{-1}$ at 1 atm pressure and 80 °C was used. b) Comparison of the Ir utilization (specific Ir demand) at 70 % LHV (~ 1.78 V cell voltage) for the 60% Ir/a.$TiO_2$ and the Umicore Elyst 75 reference catalyst at two different loadings.

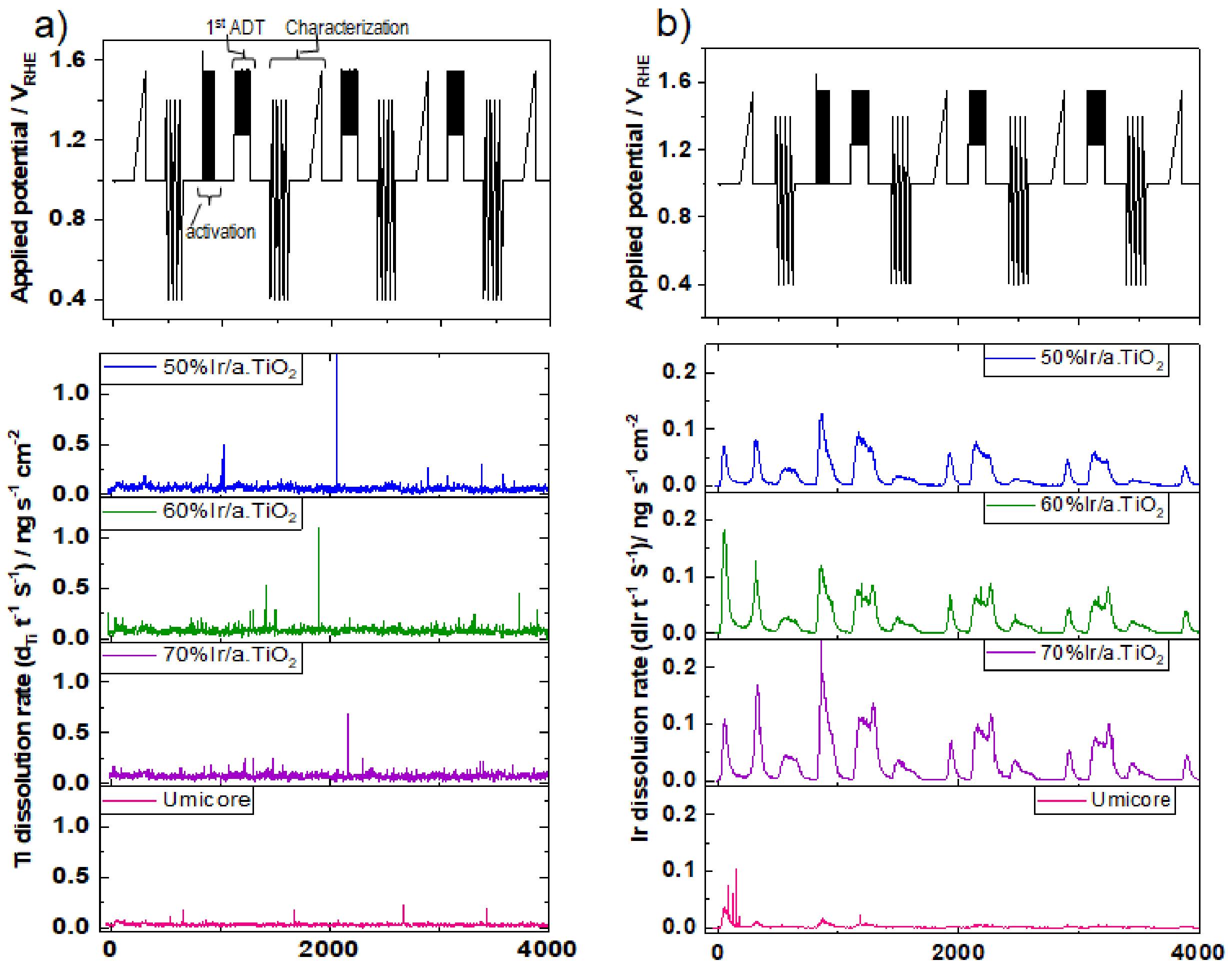


**Figure S8.** ICP – MS coupling SFC reveals dissolution profiles of Ir and Ti for four samples: 50% (blue), 60% (green), 70% Ir/a.$TiO_2$ (purple), and Umicore catalyst (pink), during ADT. A description of the applied potential program is displayed on top (a,b), which comprises three main steps: characterization, activation, and the short ADT cycles. Below, we present the dissolution profiles (in ng $s^{-1}$ $cm^{-2}$) of a) Ir and b) Ti during ADT. The measurements were conducted under the following conditions: 25°C, 0.05 M $H_2SO_4$. For the synthesized catalysts, the loading of Ir was approximately 10 µg $cm^{-2}$, for the Umicore, the Ir loading was increased to 25 µg $cm^{-2}$ to achieve a better distribution of the catalyst spots on WE.

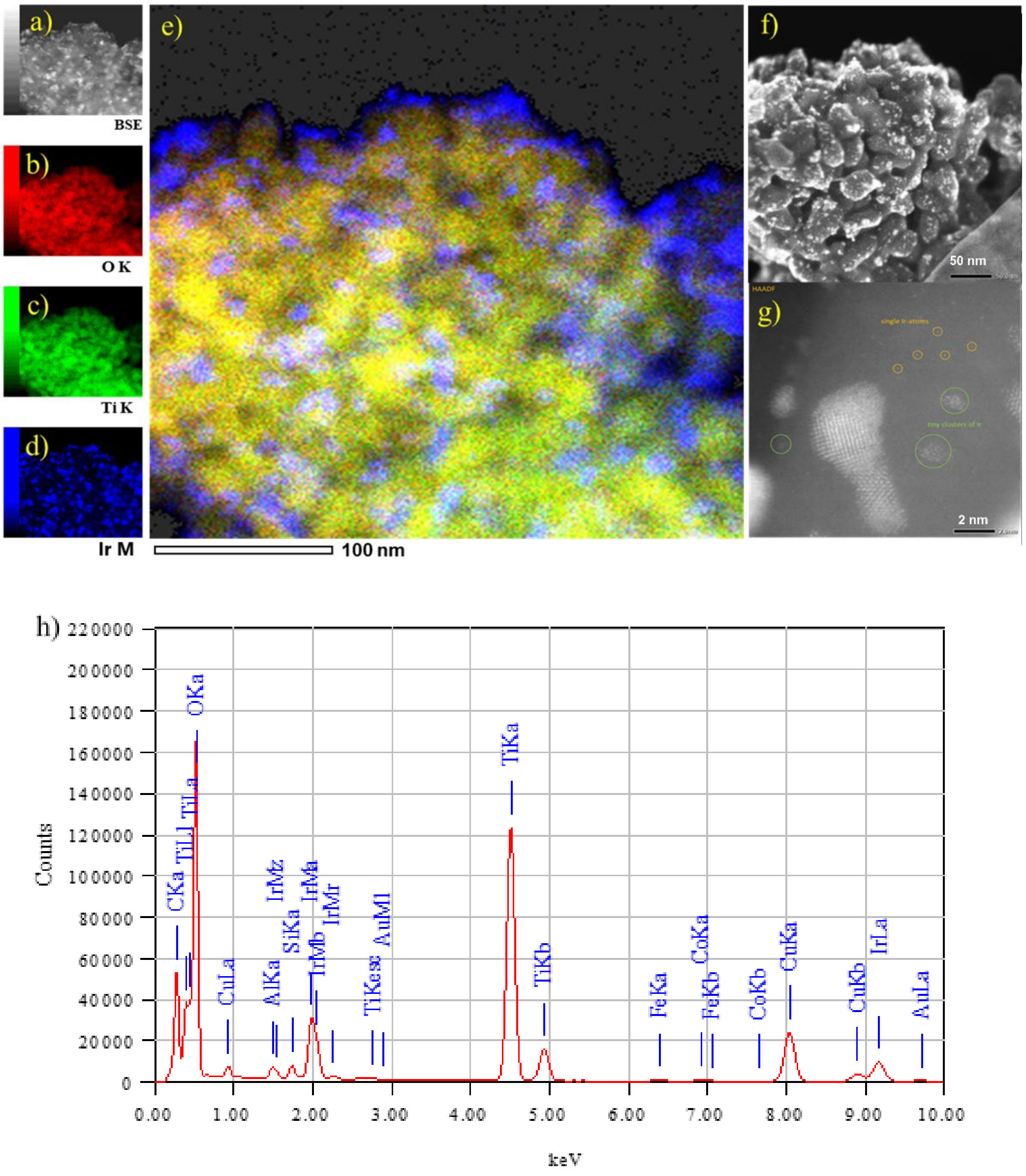


**Figure S9.** HR-STEM images and EDX mapping of Ir 60 wt % loaded catalyst supported on a.$TiO_2$ in the ***as prepared state*** (60% Ir/$TiO_2$_AP). a) Backscattered Electron Imaging (BSE) and EDX mapping of various elements, including: b) Oxygen distribution (red), c) Titanium distribution (green) and d) Iridium distribution (blue). e) Comprehensive elemental mapping using an RGB overlay. HAADF image of Ir 60% Ir/$TiO_2$_AP with a scale bar of f) 100 nm and g) 5 nm. h) Corresponding EDX spectra showing elemental maps derived from specific elemental peaks, differentiated from background noise.

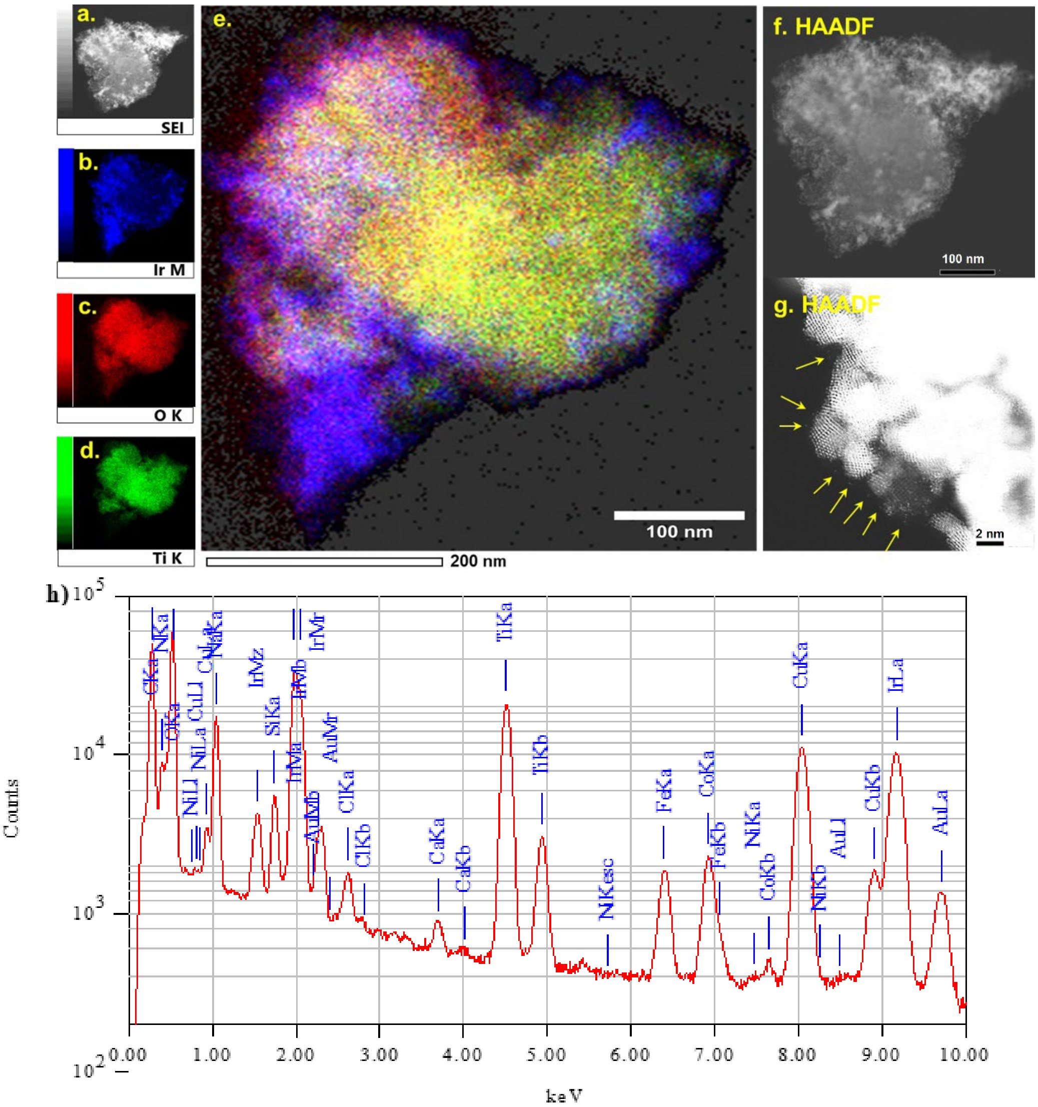


**Figure S10.** HR-STEM images and EDX mapping of the 60 wt% Ir loaded, a.$TiO_2$-supported catalyst in the ***after activation** state (*60% Ir/$TiO_2$_AA). a) Secondary Electron Imaging (SEI) and EDX mapping of various elements, including: b) Oxygen distribution (red), c) Titanium distribution (green), d) Iridium distribution (blue). e) Full elements mapping using RGB overlay. HAADF images of 60% Ir/$TiO_2$_AA with a scale bar of f) 100 nm and g) 2 nm. h) Corresponding EDX spectra showing elemental maps derived from specific elemental peaks, differentiated from background noise.

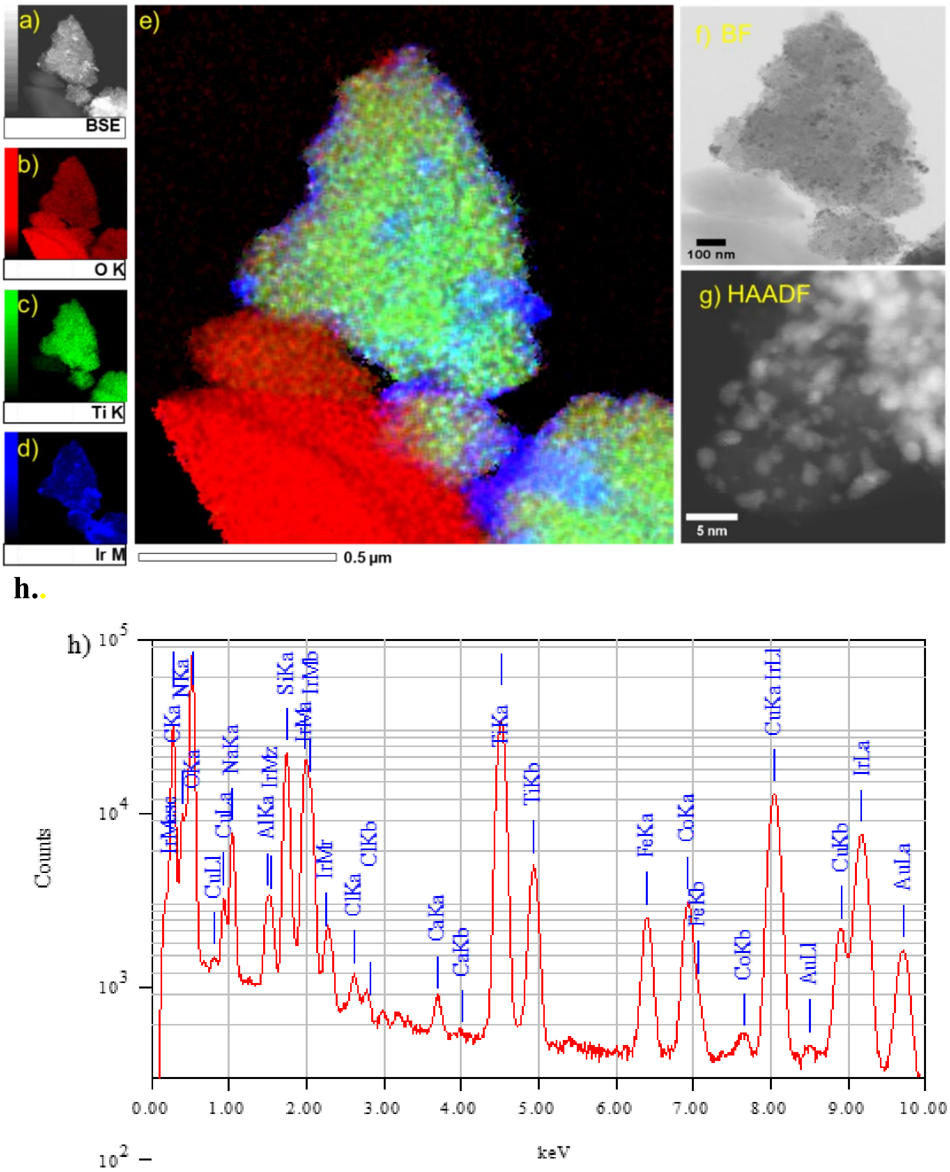


**Figure S11.** HR-STEM images and EDX mapping of the Ir 60% wt catalyst supported on a.$TiO_2$ catalyst after the ***accelerated degradation test*** (60% Ir/$TiO_2$_ADT). a) Backscattered Electron Imaging (BSE) and EDX mapping of various elements, including: b) Oxygen distribution (red), c) Titanium distribution (green) and d) Iridium distribution (blue) e) Full elements mapping using RGB overlay. f) Bright field (BF) image of 60% Ir/$TiO_2$_ADT with a scale bar of 100 nm. g) HAADF image of Ir 60% Ir/$TiO_2$_ADT with a scale bar of 5 nm. h) Corresponding EDX spectra showing elemental maps derived from specific elemental peaks, differentiated from background noise.

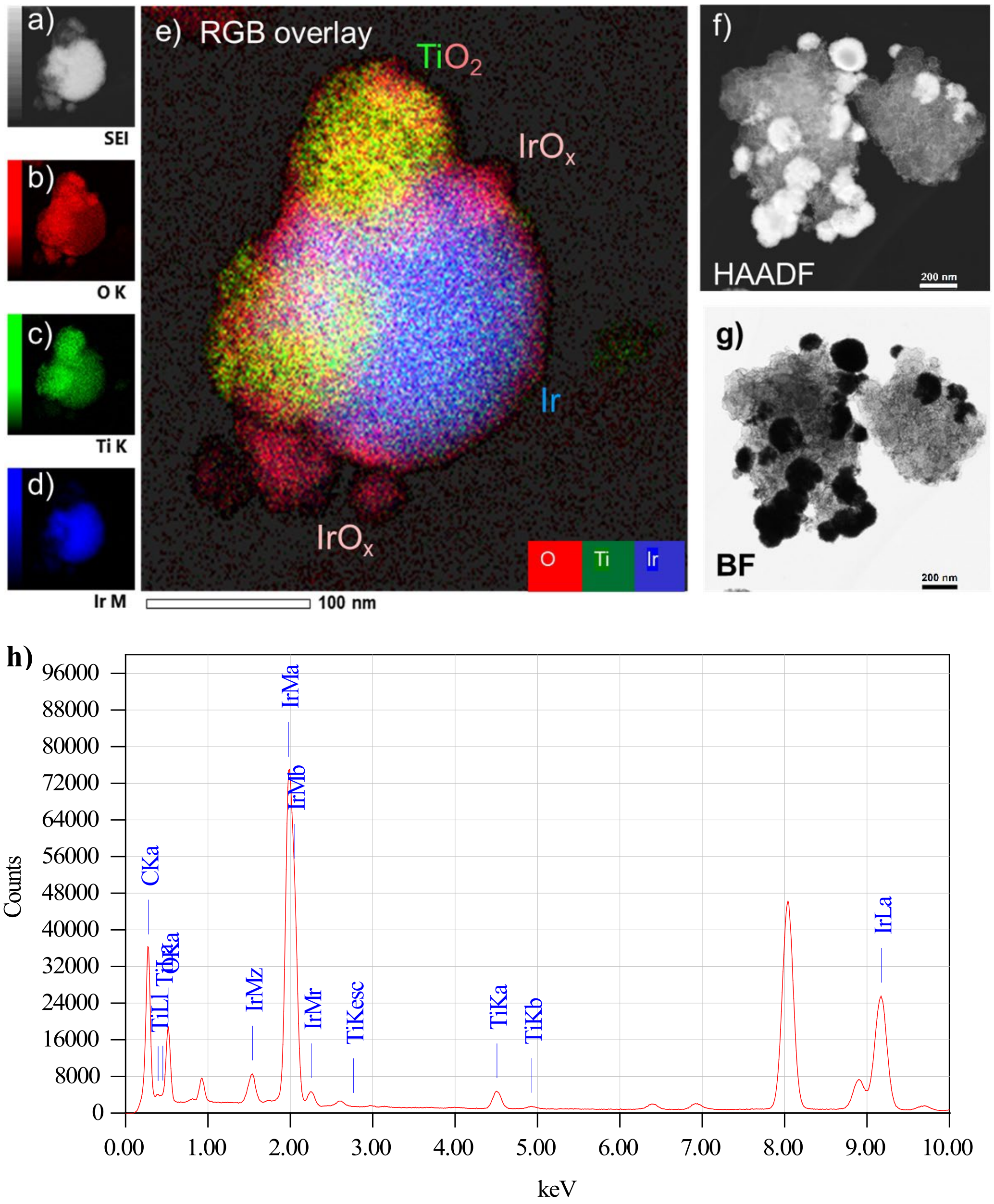


**Figure S12.** HR-STEM images and EDX mapping of the **Umicore reference catalyst**, 75 wt % of Iridium (in oxide form) supported on titanium dioxide, in the ***as prepared state***. a) Secondary Electron Imaging (SEI) ) and EDX mapping of various elements, including: b) Oxygen distribution (red), c) Titanium distribution (green) and d) Iridium distribution (blue). e) Full elements mapping using RGB overlay, f) HAADF image of Umicore catalyst, 75 wt % with a scale bar of 200 nm. g) BF image of Umicore catalyst 75 wt % with a scale bar of 200 nm. h) The corresponding EDX spectra indicate that the elemental maps were derived through the calculation of specific elemental peaks, which were discerned amidst background noise.

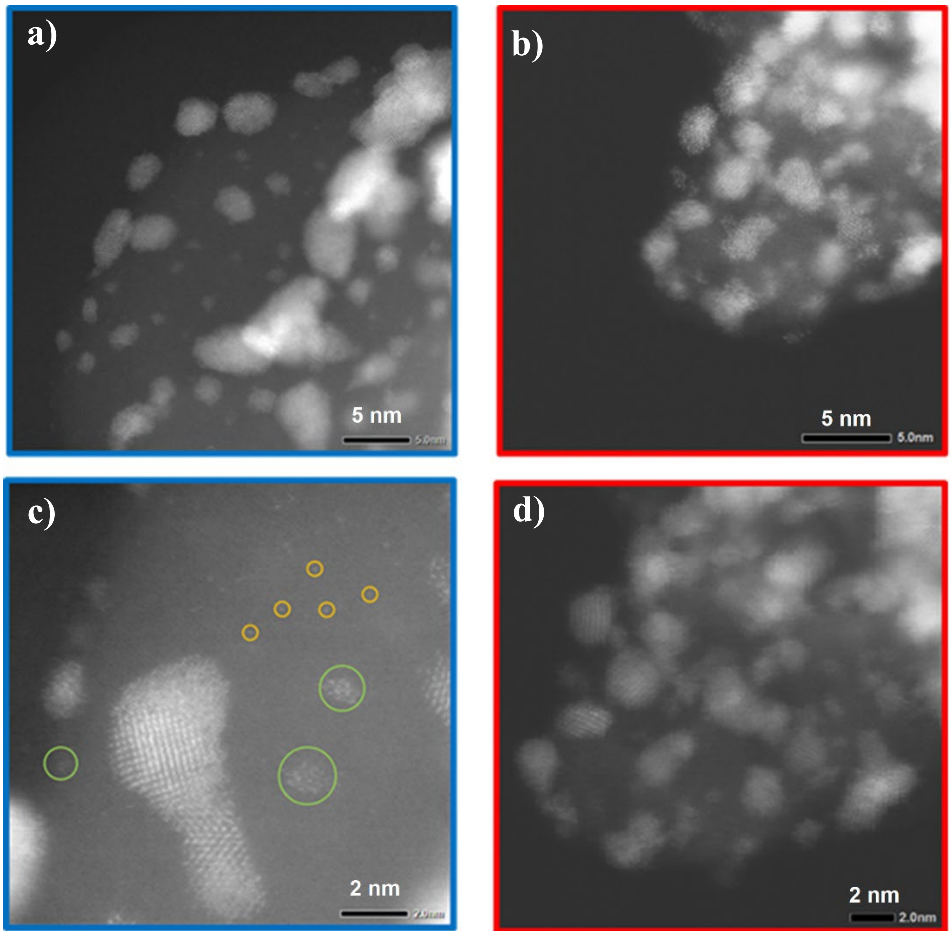


**Figure S13.** HR-STEM HAADF images of the 60 wt% Ir supported on a.$TiO_2$ (60% Ir/a.$TiO_2$) nano particle catalyst in the as-prepared state (AP) (a,c blue frame) and in the after ADT state (b,d red frames). a) 60% Ir/a.$TiO_2$_AP b) 60% Ir/a.$TiO_2$_ADT, with 5 nm scale bar; and c) 60% Ir/a.$TiO_2$_AP d) 60% Ir/a.$TiO_2$_ADT, with 2 nm scale bar.

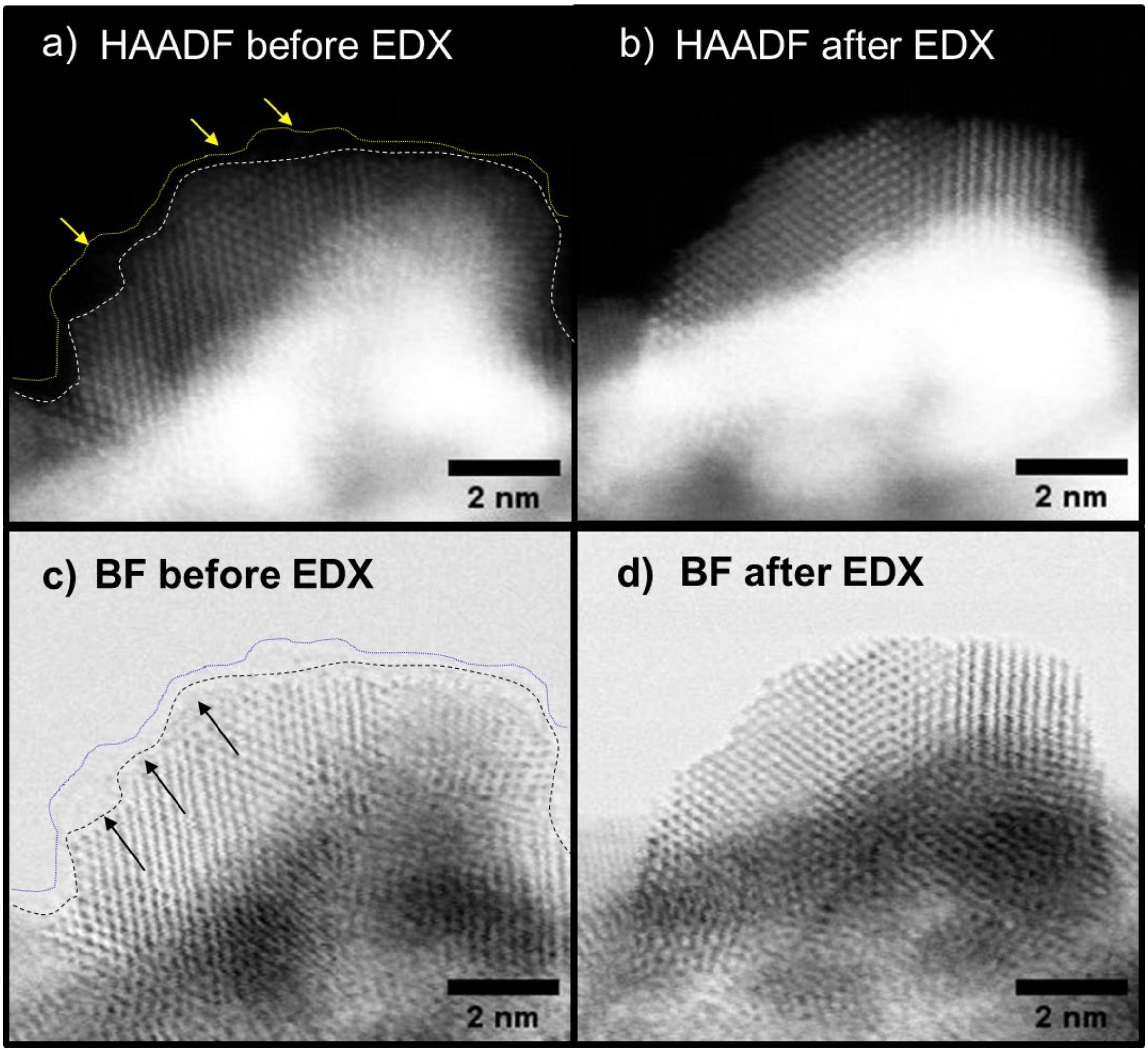


**Figure S14.** HR-STEM images of the synthesized 60% Ir/a.$TiO_2$ at the ***before (AP) and after activation (AA) state***. a) HAADF image of the catalyst surface before EDX mapping. b) HAADF image of the catalyst surface after the initial EDX mapping. c) BF image of the catalyst surface before the EDX mappings. All HR-STEM images were cropped with a 2 nm scale bar on panel (a), the white line on the HAADF image reveals the inner border of crystalline metallic Ir, while yellow arrows indicate isolated Ir atoms separated from the crystalline surface. By contrast, the BF image in panel (c) displays a thin hydrous IrO(OH)x layer, denoted by the blue curve, while black arrows and black dashed curve indicate the positions of crystalline metallic Ir. The combined image of BF and HAADF is shown in **Figure 6b**. (d) BF image of the catalyst surface taken immediately after the EDX mappings. It is noteworthy that, prior to EDX mapping, the single Ir atoms are found within the hydrous oxide layer, detached from the nanoparticles. This suggests that the observed layer is not a contamination layer resulting from sample preparation or the microscope itself but rather an intrinsic feature of the sample's pristine AP state. After performing a rapid EDX mapping at 300 kV, it was observed that within 2 minutes, the individual Ir atoms have merged with the NPs, causing the surrounding layer to disappear. Consequently, the surface

morphology undergoes changes, becoming rougher but possibly more crystalline near the surface due to beam-induced damage.

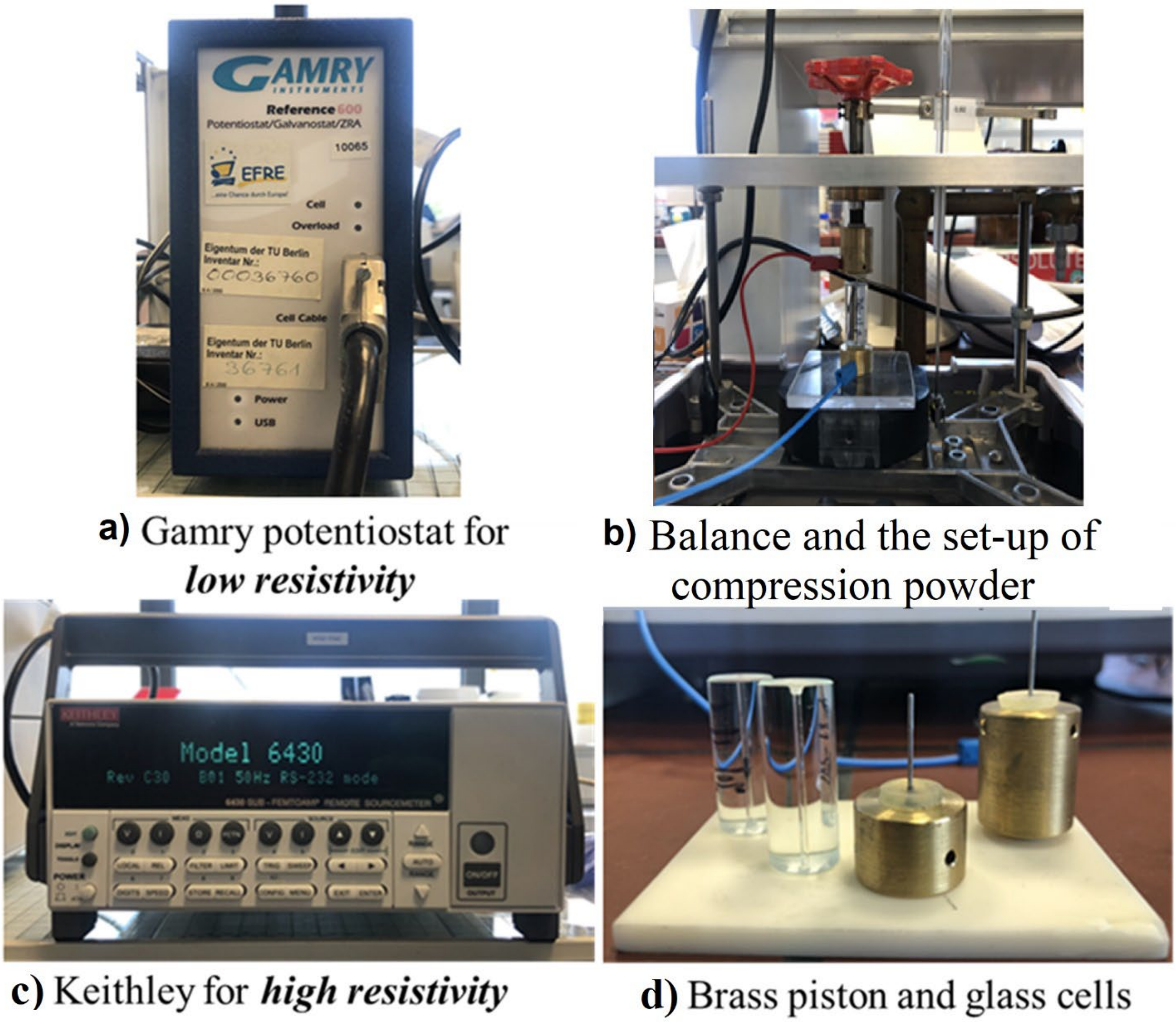


**Figure S15.** Experimental devices and set-up for conductivity measurement of compressed powders in moderation. a) The Gamry Preference 3000 Potentiostat/Galvanostat/ZRA is employed to measure the resistivity of highly conductive powders. b) The setup for compressing powder, a simplified version of the model presented in **Figure S2b**. c) Keithley 6430 Sub-Femtoamp Remote SourceMeter was applied to measure high resistivity powders, under applied 1.0 V. d) The soda-glass cylinders (on the left-hand side) and two brass pistons, comprising both the bottom part (located on the right-hand side, outside) and the top part (positioned in the center). To protect the soda-glass cylinder from compression pressure, two hard steel tips were equipped with rubber pads.

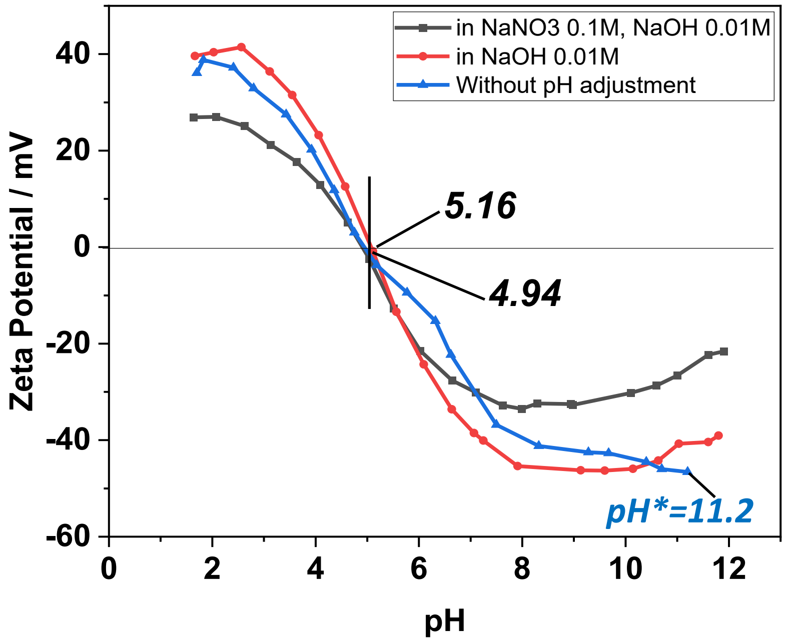


**Figure S16.** Titration curves of zeta potential versus pH for anatase titanium dioxide. the zeta potential of anatase titanium dioxide (a.$TiO_2$) is plotted against pH. The samples were dispersed in solutions of 0.1 M $NaNO_3$ with 0.01 M NaOH (black curve), 0.01 M NaOH (red curve), or without pH adjustment (blue curve) at a concentration of 0.5 mg $mL^{-1}$.

**Supplemental Note 4: Titration analysis**

Based on the titration curves spanning pH 12 to 1, the anatase titania (a.$TiO_2$) support exhibits an isoelectric point at 5.13. The original state of the a.$TiO_2$ surface is relatively acidic, with a pH value of 4.10. In other words, when dispersed in water, the surface functional groups of a.$TiO_2$ introduce protons into the suspension, resulting in a positively charged surface capable of anion adsorption. Upon dispersion in a 1M $NaNO_3$ solution, a higher concentration of ions in the surrounding atmosphere provides a more stable environment for the surface charge. Consequently, this leads to reduced surface charge variation, less fluctuation, and smaller error bars in the titration curve.

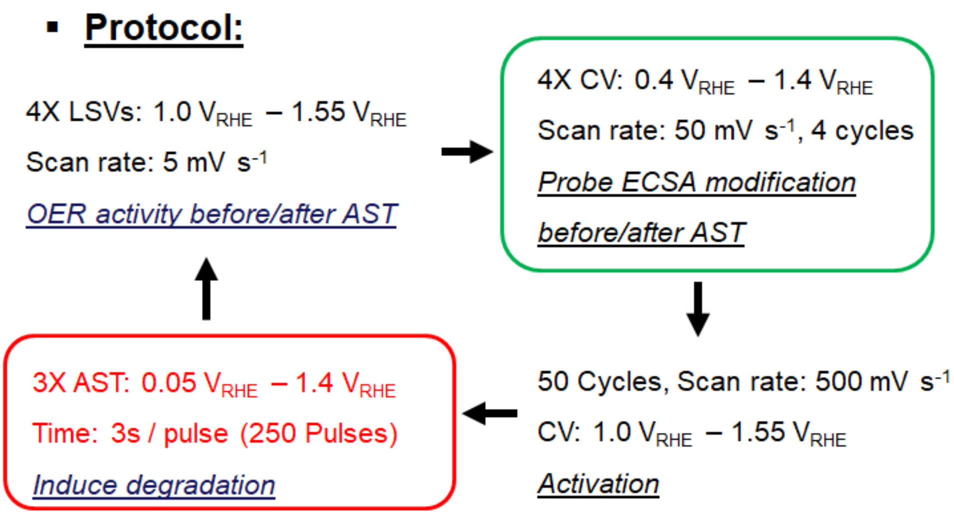


**Figure S17.** ICP – MS coupling with SFC, detailed potential program, using for SFC: characterization, activation, and short ADT.

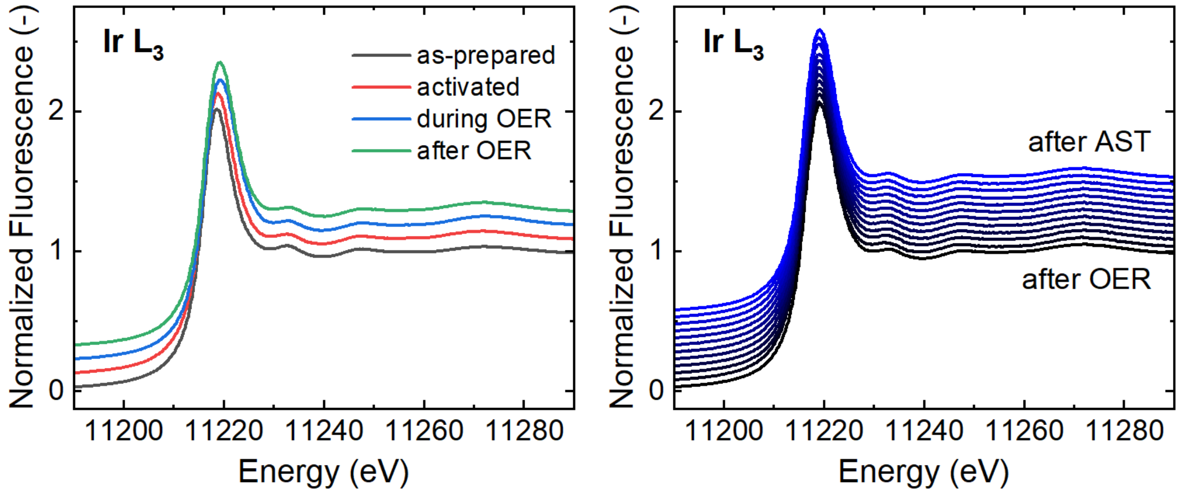


**Figure S18.** Ir $L_3$ XANES profiles of the 60% Ir/a.$TiO_2$ samples at selected electrochemical conditions (left panel) as well as during the subsequent ADT (right panel).

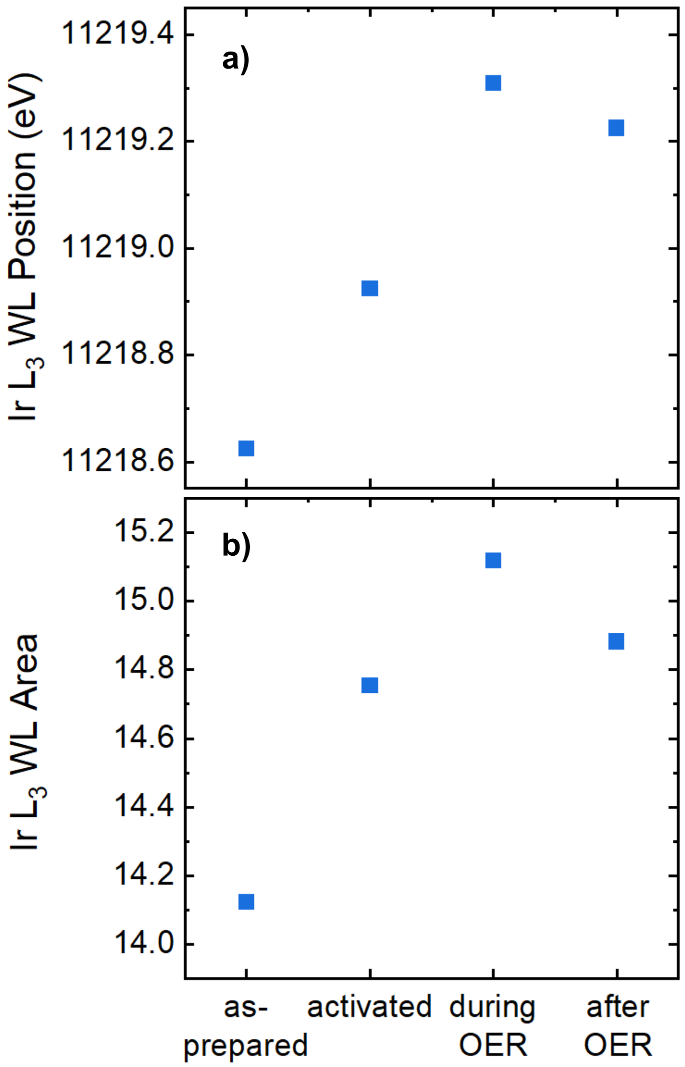


**Figure S19.** White line position a) and area b) extracted from the Ir $L_3$ XANES profiles of the 60% Ir/a.$TiO_2$ samples at selected electrochemical conditions.

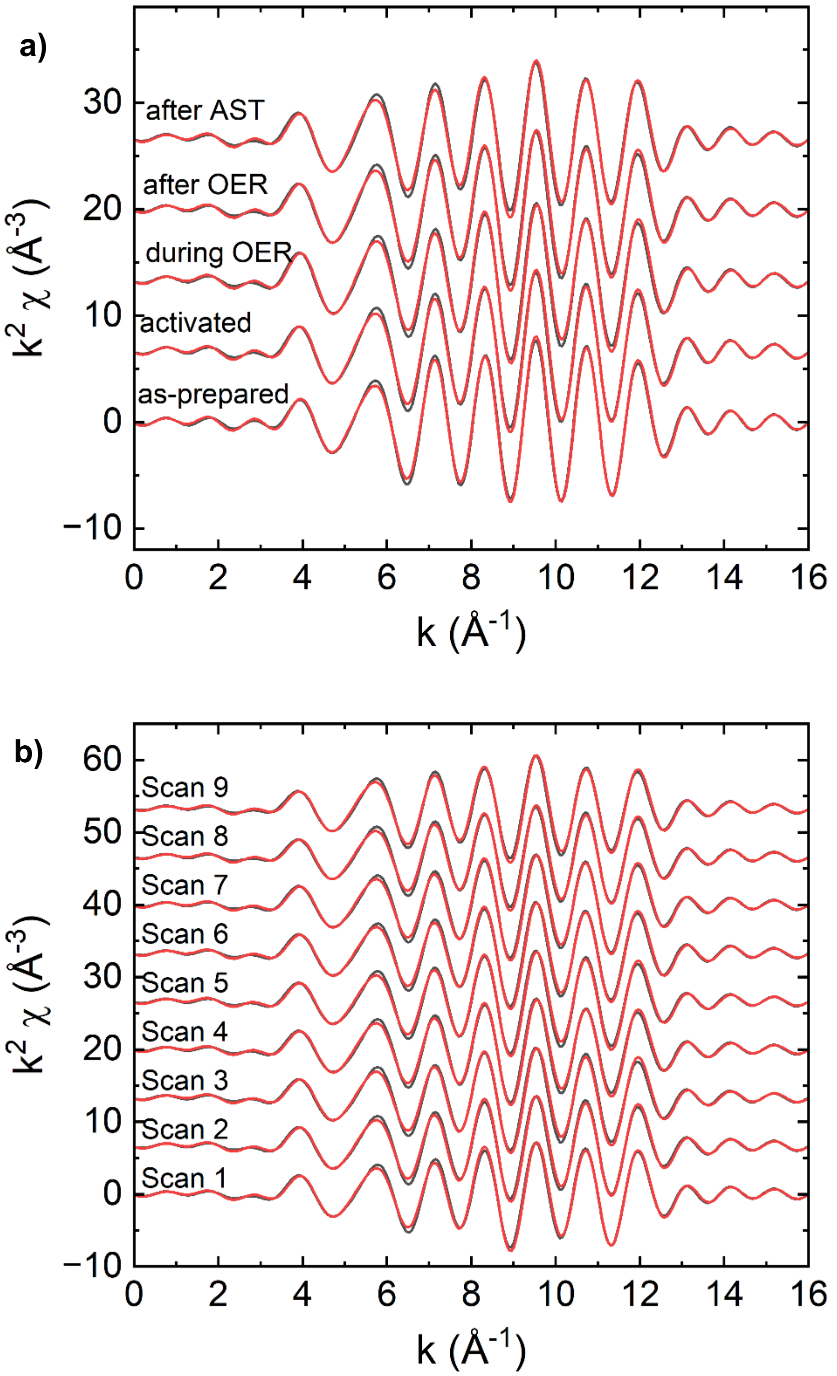


**Figure S20.** Fourier-filtered Ir $L_3$ EXAFS profiles of the 60% Ir/a.$TiO_2$ samples at selected electrochemical conditions (a) and during the accelerated stress test (b). The solid black lines show the experimental data, and the solid red lines show the fitted profiles.

**Table S1.** Packing density for various supported Ir-based catalysts.

| Sample's name | Catalyst packing density, $\rho_{cat}$, / g cm$^{-3}$ | | Iridium packing density, $\rho_{Ir}$ / g cm$^{-3}$ | |
|---|---|---|---|---|
| | 40 MPa | 1.5 MPa | 40 MPa | 1.5 MPa |
| 30% Ir/a.$TiO_2$ | 1.84 | 1.29 | 0.55 | 0.35 |
| 40% Ir/a.$TiO_2$ | 1.74 | 1.44 | 0.70 | 0.57 |
| 50% Ir/a.$TiO_2$ | 1.85 | 1.26 | 0.92 | 0.62 |
| 60% Ir/a.$TiO_2$ | 1.51 | 1.28 | 0.91 | 0.77 |
| 70% Ir/a.$TiO_2$ | 3.80 | 1.52 | 2.66 | 1.06 |
| 80% Ir/a.$TiO_2$ | 3.86 | 2.43 | 3.09 | 1.95 |
| Umicore Elyt 75% | 2.92 | 2.09 | 2.18 | 1.57 |

**Supplementary Note 5: Measurement of Packing Density Using Compression Powder Devices**

To measure packing density, a specific quantity of powder (ranging from 3 to 10 mg, depending on powder density) was weighed and carefully transferred into the core hole of a soda-glass cylinder, positioned on the bottom part of the brass piston. During the assembly of the catalyst powder, the cylinder was tapped several times to facilitate particle rearrangement and remove air gaps. The thickness of the powder inside the capillary was determined using a highly precise ruler that had been calibrated before measurement. The desired thickness fell within the range of 3 to 6 mm. After assembling the top part of the setup, a specific pressure, precisely measured using a technical balance integrated into a four-pillar manual press system, was applied to the top piston using a twist pressing wheel. The weight of the upper piston was also taken into account when calculating the compression pressure on the catalytic powder.

The packing density of Ir was calculated using the following equations:

$$\rho_{Ir} = \rho_{Cat} \cdot wt_{Ir}\% \quad (2)$$

$$\rho_{Cat} = \frac{m_{cat}}{h\,A} \quad (3)$$

Where $\rho_{cat}$ and $\rho_{Ir}$ represent the packing density of the catalyst and Ir, respectively; h is the powder thickness (in cm); and A is the cross-sectional area (in $cm^2$), considering a 0.08 cm capillary, resulting in A = 0.005 $cm^2$ for the compressed powder. The pressure was converted from kilograms (kg) to megapascals (MPa) using the following equations:

$$P\ [Pa] = P\left[\frac{N}{m^2}\right] = P\left[\frac{kg}{m.s^2}\right] = \frac{m[kg].g[m/s^2]}{A[m^2]} \tag{4}$$

$$\Leftrightarrow P[MPa] = P\left[\frac{kgf}{m^2}\right].\frac{g\ [m/s^2]}{10^6} = \frac{m[kg].10000}{A[cm^2]}.\frac{g\ [m/s^2]}{10^6} \tag{5}$$

In its simplest form:

$$P_{MPa} = \frac{m\ g}{A\ 10^2} \tag{6}$$

Where P represents compression pressure, m is the measured catalyst mass, g is the gravitational acceleration (9.807 m $s^{-2}$), and A is the cross-sectional area (measured in $cm^2$)

**Table S2.** Particle size and crystalline size of Ir NPs, the active sites and a. $TiO_2$ support materials.

| Sample's name | Particle size / nm | | Crystalline size / nm | |
|---|---|---|---|---|
| | Ir | a.$TiO_2$ | Ir | a.$TiO_2$ |
| 30% Ir/a.$TiO_2$ | 1.38 ± 0.13 | 36.32 ± 8.26 | 6.33 ± 0.56 | 10.60 ± 0.32 |
| 40% Ir/a.$TiO_2$ | 2.31 ± 0.18 | 36.32 ± 8.26 | 5.59 ± 0.64 | 10.80 ± 0.32 |
| 50% Ir/a.$TiO_2$ | 2.30 ± 0.17 | 36.32 ± 8.26 | 4.61 ± 0.77 | 10.60 ± 0.16 |
| 60% Ir/a.$TiO_2$ | 1.63 ± 0.19 | 36.32 ± 8.26 | 3.62 ± 0.98 | 10.78 ± 0.32 |
| 70% Ir/a.$TiO_2$ | 2.89 ± 0.18 | 36.32 ± 8.26 | 3.85 ± 0.92 | 11.29 ± 0.30 |
| 80% Ir/a.$TiO_2$ | 2.95 ± 0.13 | 36.32 ± 8.26 | 4.02 ± 0.88 | 11.81 ± 0.31 |

**Supplemental Note 6: Particle size and crystalline size calculation**

Particle sizes were determined using TEM images. The particle size of the pristine a.$TiO_2$ material was initially determined. Measurements were conducted for approximately five hundred particles, and then a histogram distribution and LogNormal fitting curve was applied to calculate the mean value, error and standard deviation.

Crystalline size calculation was based on the Scherrer Equation. The FWHM value ($\beta$) was determined for several selected reflections. These reflections were chosen based on their distinct intensity and position, including the Ir - (111) plane and $TiO_2$ anatase - (101) plane. The calculation involved utilizing the wavelength of the incident X-rays of Cu-K$\alpha$, with $\lambda$ = 1.5406 Å, and the Scherrer constant, denoted as K, which characterizes the particle shape. The commonly adopted value for K is 0.89.

**Table S3.** Fitting results obtained from the $k^2$-weighted EXAFS spectra for the coordination numbers (CN), the interatomic distance (R), the disorder factor ($\sigma$) and the energy shifts ($\Delta E_0$). The uncertainties are shown in brackets for the last one or two digits and reflect the standard errors calculated from the EXAFS fitting results. The $S_0^2$ values were obtained from fitting Ir metal and $IrO_2$ reference materials by fixing the coordination number to be 0.75 for $IrO_2$ and 0.78 for Ir. The fits are shown in **Supplementary Figure S19**.

| Catalyst State | Ir-O | | | Ir-Ir | | | $\Delta E_0$ (eV) | R |
|---|---|---|---|---|---|---|---|---|
| | CN (-) | R(Å) | $\sigma^2$ (Å$^2$) | CN (-) | R(Å) | $\sigma^2$ (Å$^2$) | | |
| as-prepared | 1.16(14) | 1.985(8) | 0.0058(14) | 8.2(5) | 2.698(2) | 0.0034(3) | 1.3(6) | 0.020 |
| activated | 1.42(15) | 1.961(8) | 0.0009(13) | 7.6(5) | 2.697(3) | 0.0033(3) | 0.7(6) | 0.023 |
| during OER | 1.47(16) | 1.938(8) | 0.0010(14) | 7.8(6) | 2.695(3) | 0.0037(3) | 0.7(6) | 0.025 |
| after OER | 1.52(16) | 1.954(8) | 0.0009(13) | 7.5(6) | 2.695(3) | 0.0035(3) | 0.5(6) | 0.025 |
| | | | | | | | | |
| during ADT – 1 | 1.53(16) | 1.938(8) | 0.0015(14) | 7.3(5) | 2.696(3) | 0.0033(3) | 0.6(6) | 0.024 |
| during ADT – 2 | 1.54(17) | 1.935(8) | 0.0018(15) | 7.6(6) | 2.695(3) | 0.0036(3) | 0.2(6) | 0.025 |
| during ADT – 3 | 1.49(17) | 1.935(8) | 0.0014(15) | 7.7(6) | 2.694(3) | 0.0038(3) | 0.1(6) | 0.028 |
| during ADT – 4 | 1.50(16) | 1.935(8) | 0.0012(14) | 8.0(6) | 2.693(3) | 0.0039(3) | 0.1(6) | 0.025 |
| during ADT – 5 | 1.56(17) | 1.937(8) | 0.0021(15) | 7.8(6) | 2.695(3) | 0.0038(3) | 0.18(6) | 0.02555 |
| during ADT – 6 | 1.51(15) | 1.937(8) | 0.0012(13) | 7.5(5) | 2.697(3) | 0.0035(3) | 0.5(6) | 0.024 |
| during ADT – 7 | 1.53(16) | 1.935(8) | 0.0016(13) | 7.5(5) | 2.696(3) | 0.0036(3) | 0.3(6) | 0.023 |
| during ADT – 8 | 1.59(17) | 1.951(8) | 0.0019(14) | 7.3(5) | 2.695(3) | 0.0034(3) | 0.2(6) | 0.025 |
| during ADT – 9 | 1.55(16) | 1.956(7) | 0.0009(13) | 7.5(6) | 2.695(3) | 0.0036(3) | 0.2(6) | 0.025 |
| after ADT | 1.57(16) | 1.958(8) | 0.0011(13) | 7.4(6) | 2.697(3) | 0.0035(3) | 0.7(6) | 0.026 |

## References


1. Nong, H. N.; Oh, H. S.; Reier, T.; Willinger, E.; Willinger, M. G.; Petkov, V.; Teschner, D.; Strasser, P., Oxide-supported IrNiO(x) core-shell particles as efficient, cost-effective, and stable catalysts for electrochemical water splitting. *Angew Chem Int Ed Engl* **2015,** *54* (10), 2975-9.
2. Nong, H. N.; Gan, L.; Willinger, E.; Teschner, D.; Strasser, P., IrOx core-shell nanocatalysts for cost- and energy-efficient electrochemical water splitting. *Chem. Sci.* **2014,** *5* (8), 2955-2963.
3. Reier, T.; Teschner, D.; Lunkenbein, T.; Bergmann, A.; Selve, S.; Kraehnert, R.; Schlögl, R.; Strasser, P., Electrocatalytic Oxygen Evolution on Iridium Oxide: Uncovering Catalyst-Substrate Interactions and Active Iridium Oxide Species. **2014**.
4. Spöri, C.; Brand, C.; Kroschel, M.; Strasser, P., Accelerated Degradation Protocols for Iridium-Based Oxygen Evolving Catalysts in Water Splitting Devices. *Journal of the Electrochemical Society* **2021,** *168*, 034508.
5. Espinola, A.; Miguel, P. M.; Salles, M. R.; Pinto, A. R., Electrical properties of carbons—resistance of powder materials. *Carbon* **1986,** *24* (3), 337-341.
6. Karimi, F.; Peppley, B. A., Metal Carbide and Oxide Supports for Iridium-Based Oxygen Evolution Reaction Electrocatalysts for Polymer-Electrolyte-Membrane Water Electrolysis. *Electrochimica Acta* **2017,** *246*, 654-670.
7. Munnik, P.; de Jongh, P. E.; de Jong, K. P., Recent Developments in the Synthesis of Supported Catalysts. *Chemical Reviews* **2015,** *115* (14), 6687-6718.
8. Moreau, F.; Bond, G. C., Preparation and reactivation of Au/TiO2 catalysts. *Catalysis Today* **2007,** *122* (3), 260-265.
9. Zanella, R.; Delannoy, L.; Louis, C., Mechanism of deposition of gold precursors onto TiO2 during the preparation by cation adsorption and deposition–precipitation with NaOH and urea. *Applied Catalysis A: General* **2005,** *291* (1), 62-72.
